# Vactosertib, a novel TGF-β1 type I receptor kinase inhibitor, improves T-cell fitness: a single-arm, phase 1b trial in relapsed/refractory multiple myeloma

**DOI:** 10.21203/rs.3.rs-3112163/v1

**Published:** 2023-07-17

**Authors:** Ehsan Malek, Priyanka S. Rana, Muthulekha Swamydas, Michael Daunov, Masaru Miyagi, Elena Murphy, James J. Ignatz-Hoover, Leland Metheny, Kim Seong Jin, James J. Driscoll

**Affiliations:** 1 Adult Hematologic Malignancies & Stem Cell Transplant Section, Seidman Cancer Center, University Hospitals Cleveland Medical Center, Cleveland, OH; 2 Division of Hematology Oncology, Case Western Reserve University School of Medicine, Cleveland, OH; 3 Case Comprehensive Cancer Center, Case Western Reserve University, Cleveland, OH; 4 Department of Pharmacology, Case Western Reserve University School of Medicine, Cleveland, OH; 5 Center for Proteomics and Bioinformatics, Case Western Reserve University School of Medicine, Cleveland, OH; 6 Department of Biochemistry, Case Western Reserve University School of Medicine, Cleveland, OH; 7 Medpacto Inc., Seoul, Republic of Korea

**Keywords:** Multiple myeloma, TGF-β receptor kinase inhibitor, immunosuppression, cytotoxic T-lymphocyte, T-cell fitness

## Abstract

Functional blockade of the transforming growth factor-beta (TGF-β) signaling pathway improves the efficacy of cytotoxic and immunotherapies. We conducted a phase 1b study to determine the safety, efficacy, and maximal tolerated dose (200 mg po bid) of the potent, orally-available TGF-β type I receptor kinase inhibitor vactosertib in relapsed and/or refractory multiple myeloma patients who had received ≥2 lines of chemoimmunotherapy. Vactosertib combined with pomalidomide was well-tolerated at all doses, had a manageable adverse event profile and induced durable responses with 80% progression-free survival (PFS-6) at 6 months, while pomalidomide alone historically achieved 20% PFS-6. Following treatment, the immunosuppressive marker PD-1 expression was reduced on patient CD8^+^ T-cells. Following *ex vivo* treatment, vactosertib decreased PD-1 expression on patient CD138^+^ cells, reduced PD-L1/PD-L2 on patient CD138^+^ cells and enhanced the anti-myeloma activity of autologous T-cells. Taken together, vactosertib is a safe immunotherapy that modulates the T-cell immunophenotype to reinvigorate T-cell fitness. Multiple myeloma (MM) is a genetically heterogeneous hematologic malignancy characterized by the excessive proliferation of clonal plasma cells (1, 2). MM remains mostly incurable but a small group of patients can achieve long-term remission (3). Treatment of MM presents unique challenges due to the complex molecular pathophysiology and genetic heterogeneity (4, 5). Given that MM is the second most common blood cancer characterized by cycles of remission and relapse, the development of new therapeutic modalities is crucial (6, 7). The prognosis for MM patients has improved substantially over the past two decades with the development of more effective therapeutics, e.g., proteasome inhibitors, and regimens that demonstrate greater anti-tumor activity (8–10). The management of RRMM represents a vital aspect of the overall care for patients with disease and a critical area of ongoing scientific and clinical research (10–12).

Over the past two decades, the myeloma community has witnessed the success of immunomodulatory drugs (IMiDs), monoclonal antibodies, chimeric antigen receptor (CAR) T-cells and bispecific T-cell engagers (BiTEs) that broke the *circulus vitiosus* of tumor-induced immunosuppression to reengage the immune system (13, 14). IMiDs are widely used as both primary therapy and maintenance therapy and resistance to prior lenalidomide (Revlimid) or pomalidomide (Pomalyst) treatment is a major patient classification feature. However, nearly all patients ultimately relapse through molecular mechanisms that remain elusive, even those who achieve a minimal residual disease (MRD)^neg^ status and a complete remission to initial therapy.

The TGF-β1 signaling pathway promotes cancer progression by concomitantly enhancing tumor growth, drug resistance and metastases while inhibiting the host immune response (15, 16). TGF-β1 signals through type I (TβRI) and type II (TβRII) receptors by directly phosphorylating the downstream transcription factors Smad2/Smad3 (17). While malignant plasma cells are not known to harbor mutations in the TGF-β pathway, increased TGF-β1 secreted by myeloma and stromal cells impairs immunosurveillance and promotes disease progression. An elevated level of TGF-β1 in MM patient sera correlates with drug resistance, tumor progression and poor prognosis (18). Since TGF-β1 promotes myeloma growth and drug resistance, we hypothesized that inhibition of TGF-β1 signaling represents a viable strategy to treat RRMM. Vactosertib (TEW-7197, Medpacto, Seoul, S. Korea) is a selective inhibitor of the serine/threonine TGF-β type I receptor kinase (ALK4/ALK5) that suppresses tumor progression and metastatic growth in preclinical cancer models (19, 20). While the effects of TGF-β1 on tumor cells are varied and context-specific, its role in immune evasion within the tumor microenvironment (TME) appears similar across cancers since TGF-β1 is a central mediator of immune tolerance (21–24). Vactosertib exhibits tumor intrinsic effects and also possesses activity against numerous immune cell types (25, 26). These observations led us to determine the safety and efficacy of vactosertib and to test the hypothesis that disrupting the TGF-β1 pathway can overcome immunosuppression.

## METHODS

### Study Design and participants.

**Figure 1.**
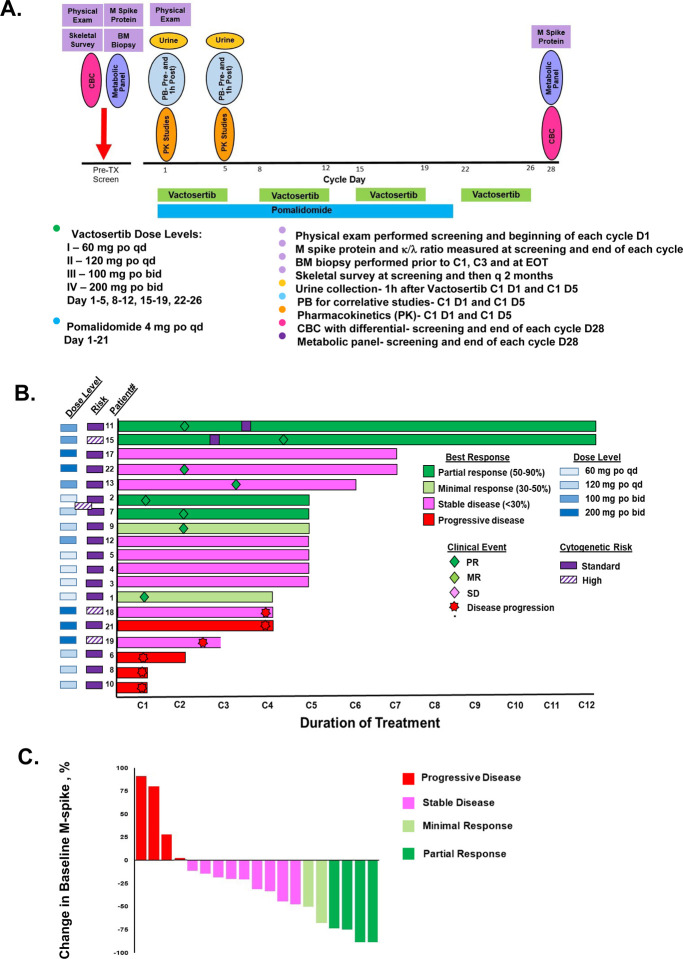
Trial design, Swimmer’s plot and Waterfall plot to evaluate the response of patients with RRMM to vactosertib combined with pomalidomide. **A.** Vactosertib and pomalidomide dosing schedule and vactosertib dose levels for the phase 1b dose escalation cohort. Also shown is the schedule for clinical and laboratory testing. **B.** Shown is individual trajectories and outcomes over duration in the 19 patients that received full-dose treatment. **C.** Shown is the maximal change in M-spike value for the same 19 patients. Two patients (#14 and #16) were excluded from response and outcome analysis because of withdrawn consent or grade 4 toxicity.

In this single-centre, single-arm, phase 1b study, we enrolled patients aged ≥18 years from the Seidman Cancer Center (Cleveland, OH, USA) who had a diagnosis of RRMM, measurable disease and adequate organ function. Patients had received ≥2 previous lines of therapy including IMiD and proteasome inhibitor (see Appendix I for full protocol). All patients were enrolled on the IRB-approved protocol, following good practice guidance and protected under the Helsinki Declaration. Patients were refractory to their last line of therapy and had an Eastern Cooperative Oncology Group (ECOG) performance status of ≤2. Participants received vactosertib in combination with pomalidomide. The trial was designed and performed as a classical 3+3 dose escalation phase I study. Since dose limiting toxicities were not observed at any dose level, there was no need for cohort extension. During each 28 day treatment cycle, vactosertib was administered for five consecutive days weekly for four weeks (days 1–5, 8–12, 15–19, 22–26) with a two day rest period included each week (days 6–7, 13–14, 20–21 and 27, 28) (Fig. 1A). Vactosertib was given at dose levels of 60 mg qd, 120 mg qd, 100 bid and 200 mg bid *per* the treatment schedule. Pomalidomide was administered at 4 mg po qd for 21 consecutive days at the initiation of each 28-day cycle. Primary endpoints were the safety and tolerability profile of vactosertib and pomalidomide and to determine the maximum tolerated dose (MTD). The primary analysis was done *per* protocol, in the all-treated and efficacy-evaluable population. Secondary endpoints were clinical benefit rate [complete response (CR), very good partial response (VGPR), partial response (PR), minimal response (MR), and stable disease (SD)] based on International Myeloma Working Group (IMWG) defined criteria, response duration, pharmacokinetics (PK) and progression-free survival (PFS). The effect of vactosertib and pomalidomide on TGF-β1 and interleukin (IL)-6 levels, CD138^+^, CD38^+^ cells, CD4^+^ CD8^+^, NK cells, macrophages, monocytes, and myeloid-derived suppressor cells from patient bone marrow (BM) was determined. The study is completed but survival follow-up is ongoing. The study is registered with ClinicalTrials.gov., NCT03143985.

### Inclusion and Exclusion criteria.

All inclusion and exclusion criteria are listed in Appendix A.

### Procedures.

All patients received pomalidomide orally on days 1–21 of a 28-day cycle as indicated in the treatment cycle, until progression or intolerance, for up to 24 months on protocol. Thromboprophylaxis was required at a minimum of 81 mg of aspirin. Response assessment were made using laboratory tests specified by the IMWG Uniform Response Criteria on clinic visits on day one of indicated cycles and on day one of every third cycle starting with cycle 2. Patients had BM aspirates and biopsy samples taken at baseline and at progression or end of study. Patients were removed from the study if they had disease progression, withdrew consent, had a diagnosis of new malignancy requiring systemic therapy, or developed unacceptable toxic effects. Adverse events were monitored during clinic visits, hospitalizations, and *via* documented patient-initiated phone conversations, and categorized *per* the Common Terminology Criteria for Adverse Events (CTACE, version 4.0). Adverse events were considered serious if they resulted in death, were life-threatening, required unplanned hospitalization, or resulted in persistent or significant incapacity or disruption of ability to conduct normal life functions. Intra-patient dose modification, *per* protocol, was not performed for medication-related toxicity or adverse events.

### Outcomes.

The primary endpoint was the safety of vactosertib combined with pomalidomide as defined by National Cancer Institute (NCI) CTACE criteria as the time of enrollment until disease progression, serious adverse event or death. Secondary endpoints were to determine the effect of vactosertib and pomalidomide on PFS at 6 months (PFS-6), response rates, PK properties of vactosertib and effects of vactosertib on patient cytokines, tumor cells and immune cells. PFS-6 was determined as previous.

### Statistical analysis.

PFS at six months and event-free survival were estimated using the KaplanMeier method and 95% CIs were calculated. We estimated associations of PFS with a univariate Cox proportional hazards model. Analyses were performed using RStudio version 1.2.5.

### Measurement of cell viability.

Cell viability was determined using the XTT assay. Briefly, 5,000 cells were added to 96 well plates in a volume of 250 uL in complete RPMI media that lacked phenol red. Drugs were added at indicated concentrations and plates incubated under standard conditions for 72 h. Activated-XTT reagent (50 μL) was then added, plates were incubated for 3 h at 37°C and absorbance measured at 475nM.

### Isolation of CD138^+^ and CD8^+^ cells.

Fresh BM samples were obtained from patients at indicated times and BM mononuclear cell (BMMC) cells isolated after RBC lysis and density centrifugation. CD138^+^ cells were isolated from the BMMC fraction using the EasySep^™^ human CD138 positive selection kit II (StemCell Technology, Cambridge, MA). CD8^+^ T-cells were isolated from the BMMC fraction using the EasySep direct human CD8^+^ T-cell isolation kit (StemCell Technology). The T-cell fraction was enriched from the BMMC fraction and free of CD138^+^ BM cells.

### Flow cytometry.

Healthy human T-cells and MM patient T-cells (10^6^/assay) were washed and resuspended in 100 μL FACS staining buffer with markers and respective isotype controls at room temperature for 15 min followed by three washes. MM patient CD138^+^ cells (10^5^/assay) were washed and resuspended in 100 μL FACS staining buffer with markers and respective isotype controls at room temperature for 15 min followed by three washes. All samples were analyzed on an AttuneNxT flow cytometer (ThermoFisher Scientific, Waltham, MA). Data were analyzed using FlowJo v10.8.1 software (Ashland, OR).

### Role of the funding source.

The investigators initiated and designed the trial, collected and had full and unrestricted access to the data, and performed the final analysis. Medpacto provided study drug, but had no role in study design, data collection, analysis, interpretation, or writing the report.

## RESULTS

Between July 1, 2017 and June 30, 2022, 21 patients were enrolled in the phase 1b dose escalation cohort and received vactosertib plus pomalidomide as indicated (Fig. 1A). The median patient age was 68 years (range 55–77), three patients (14%) were >75 years of age and five patients (24%) had high-risk disease (Table 1). The majority of patients were White (N=13, 62%), 7 (33%) self-identified as Black/African-American and most were male (N=13, 62%). All patients had previously received a proteasome inhibitor and an IMiD. Most patients had an ECOG score of one (N=15, 71%) while six had a score of 0 (N=6, 29%). Most patients had IgG-specific disease (N=15, 71%), five had IgA disease (24%) and one had light chain disease light chain (5%). Three patients (14%) had received one prior line of therapy, 12 (57%) had received two prior lines of therapy, four (19%) had received three lines of therapy and two (10%) had received four prior lines of therapy. Median prior lines of therapy were two. Most patients also had undergone autologous stem cell transplant (ASCT) (N=14; 67%). Best responses to prior treatment were SD (N=1; 5%); PR (N=7; 33%); VGPR (N=5; 24%); CR (N=6; 29%) and PD (N=2; 10%).

Patients were treated with vactosertib at one of four dose levels (Fig. 1A). Dose level I was 60 mg po qd; level II was 120 mg po qd; level III 100 mg po bid; and level IV was 200 mg po bid. Vactosertib was administered on days 1–5, 8–12, 15–19, 22–26 of 28-day cycles. Patients were simultaneously treated with pomalidomide administered at four mg po qd on days 1–21. The number of patients enrolled *per* dose level was six at 60 mg qd, six at 120 mg qd, four at 100 mg bid, and five at 200 mg bid. Twenty-one patients were enrolled and nineteen were reviewed in the safety, preliminary activity analyses and included in the PK and immunologic studies. One patient self-withdrew from the study and one patient was removed from due to grade 4 hepatotoxicity. However, that patient did not receive an adequate dose of vactosertib (≥75% of a cumulative dose of vactosertib cycle) to be considered for efficacy and toxicity studies.

The summary of grade 1–4 adverse events is shown (Table 2). Dose-limiting toxicities in the overall patient population were limited following oral administration of vactosertib at all 4 dose levels as well as in the highest vactosertib dose cohort tested (200 mg po bid). A single grade 4 elevated bilirubin (5%) and a single grade 4 elevated lipase (5%) were observed as well as four grade 4 events of decreased neutrophils (19%). The frequency of other grade 3 or worse non-hematological treatment-emergent adverse events was generally low. A grade 3 decrease in neutrophil, white blood cell, and lymphocyte was observed in 11 (52%), seven (33%), and six (29%), respectively. The most common adverse events of grade 3 were clinically manageable. A serious treatment-emergent event (grade 4 hepatotoxicity) was reported in 1 patient (5%). There were no deaths from adverse events possibly related to treatment within 30/90 days of treatment and one death from myeloma progression. Therefore, 200 mg po bid was defined as the MTD.

Four patients demonstrated a PR, two had a MR, nine had SD and four had disease progression (Fig. 1B). Two patients received over 12 cycles of vactosertib plus pomalidomide. The median time to response for all patients and the responders eight weeks and median duration of response was ~12 weeks. The best overall response for individual patients to treatment is shown (Fig. 1C). Waterfall plot indicated that 15 of 19 patients exhibited clinical benefit. Clinical activity was observed across all four dose levels; however, deeper responses were observed at the higher drug dose levels. Four patients exhibited a partial response, two had minimal response, nine had stable disease and four had progressive disease (Table 3).

PK properties of vactosertib following oral administration were well-characterized by a two-compartment model with parallel first-order and Michaelis-Menten elimination and approximated target-mediated drug disposition kinetics (Supp. Table 1). T_*max*_ at 100 mg was observed at 1.3 +/−0.5 h, and at 200 mg at observed 1.5 +/−1.0 h. The half-life (t_1/2_) after 100 mg administration was 3.5 +/−0.4 h and after administration of 200 mg administration was 3.3 +/− 1.3 h. The AUC_obs_ was 6175.5 +/−1349.7 ng/ml.h after oral administration of 100 mg vactosertib and 4251.8 +/−1307.6 ng/ml.h after oral administration of 200 mg vactosertib. Vactosertib PKs were previously assessed by nonlinear mixed-effects modelling of plasma concentration-time data obtained from a phase 1 trial conducted in patients with advanced solid tumors (20, 27). The halflife of 3.5 h observed here was comparable to the value (3.0 h) determined in a phase I trial of vactosertib in combination with paclitaxel as a second-line treatment in metastatic gastric adenocarcinoma (28).

**Figure 2.**
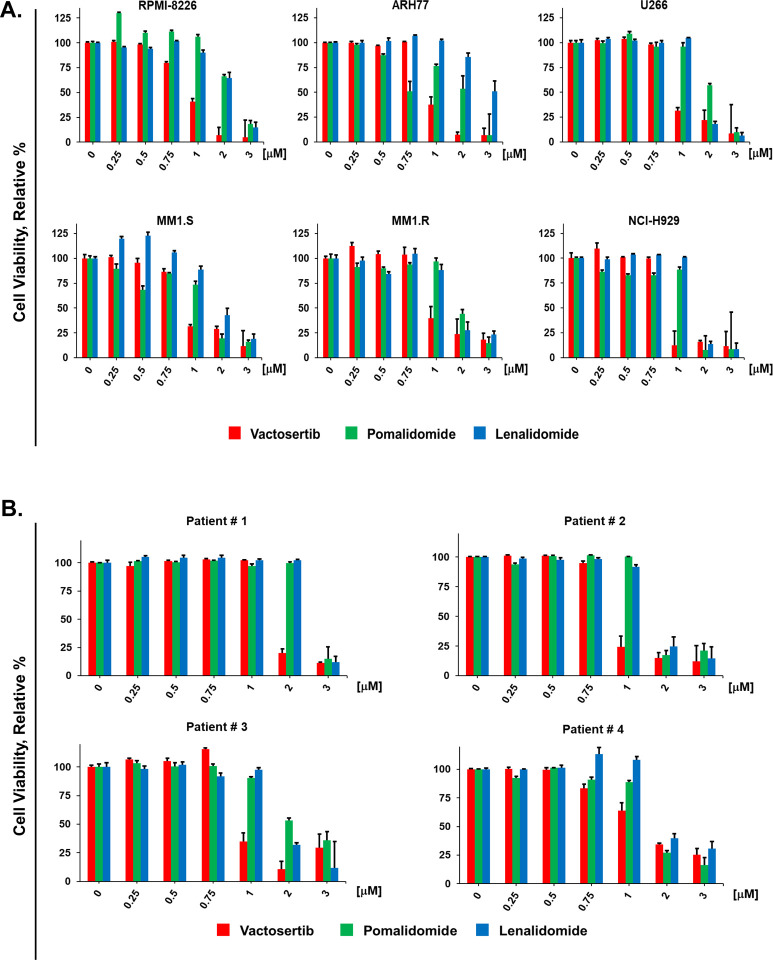
Tumor intrinsic effect of vactosertib on MM patient CD138^+^ cells and MM cell lines. **A.** Effect of vactosertib, pomalidomide, and lenalidomide on MMCLs. **B.** Effect of vactosertib, pomalidomide, and lenalidomide on CD138^+^ cells isolated from MM patient BM samples. CD138^+^ cells were isolated using the positive selection method. Patient CD138^+^ cells or MMCLs (5,000 cells/well) were incubated in 96-well plates with each drug at the indicated concentration for 72 h. The effect of drugs on cell viability was determined using the XTT assay (29). Bioassays were performed in triplicate. Error bars represent the SE of the mean.

**Figure 3.**
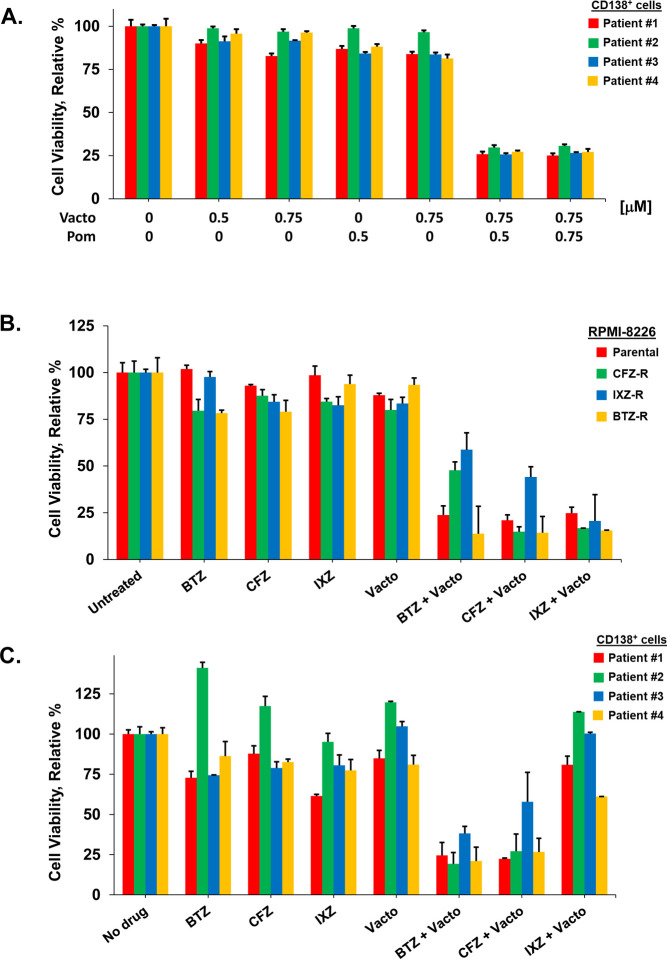
Effect of vactosertib on MM patient CD138^+^ cells and MM cell lines resistant to proteasome inhibitors. **A.** Synergistic effect of vactosertib combined with pomalidomide on drug resistant MM cells. MM patient CD138^+^ cells were incubated with vactosertib, pomalidomide or both for 72 h. **B.** Effect of vactosertib on MM cells resistant to proteasome inhibitors. RPMI8226 cells resistant to bortezomib, carfilzomib and ixazomib were generated as described (Supp. Fig. 4). Cells were incubated alone, with each proteasome inhibitor, vactosertib or each proteasome inhibitor combined with vactosertib. **C.** Effect of vactosertib on MM patient CD138^+^ cells. Cells were incubated alone or with proteasome inhibitors, vactosertib or both. Viability was determined using the XTT assay and performed in triplicate. Error bars represent the SE of the mean.

To probe the tumor intrinsic anti-myeloma activity of vactosertib, we first determined the relative effect of vactosertib compared to the IMiDs pomalidomide and lenalidomide (Fig. 2A, B). Six different MM cell lines (MMCLs) and CD138^+^ cells isolated from BM samples of four MM patients were incubated with each agent at the indicated concentrations. Cell viability determined using the XTT assay (29). At concentrations ≥750 nM, vactosertib reduced the viability of MMCLs and patient CD138^+^ cells in a dose-dependent manner. The effect of vactosertib was greater than that observed with either lenalidomide or pomalidomide. MMCLs and patient CD138^+^ cells were then treated with vactosertib in combination with pomalidomide, and importantly, vactosertib with pomalidomide synergistically reduced myeloma viability (Fig. 3A).

The proteasome inhibitor bortezomib is included in standard-of-care regimens for newly diagnosed MM (NDMM) in transplant-eligible and -ineligible patients. However, nearly all patients inevitably develop drug resistance, the relapse rate is high and RRMM is particularly resistant to conventional chemotherapy. To probe the effect of vactosertib on drug resistant cells, RPMI8226 cells were exposed to progressively escalated doses of bortezomib, carfilzomib and ixazomib. Dose-dependent drug testing indicated that RPMI8226 cells resistant to proteasome inhibitors were cross-resistant to treatment with the other two proteasome inhibitors (Supp. Fig. 4). Importantly, the drug resistant MMCLs were equally sensitive to vactosertib relative to parental RPMI8226 cells (Fig. 3B). Moreover, vactosertib and a proteasome inhibitor co-treatment of drug resistant cells or CD138^+^ cells from RRMM patients reduced cell viability.

**Figure 4.**
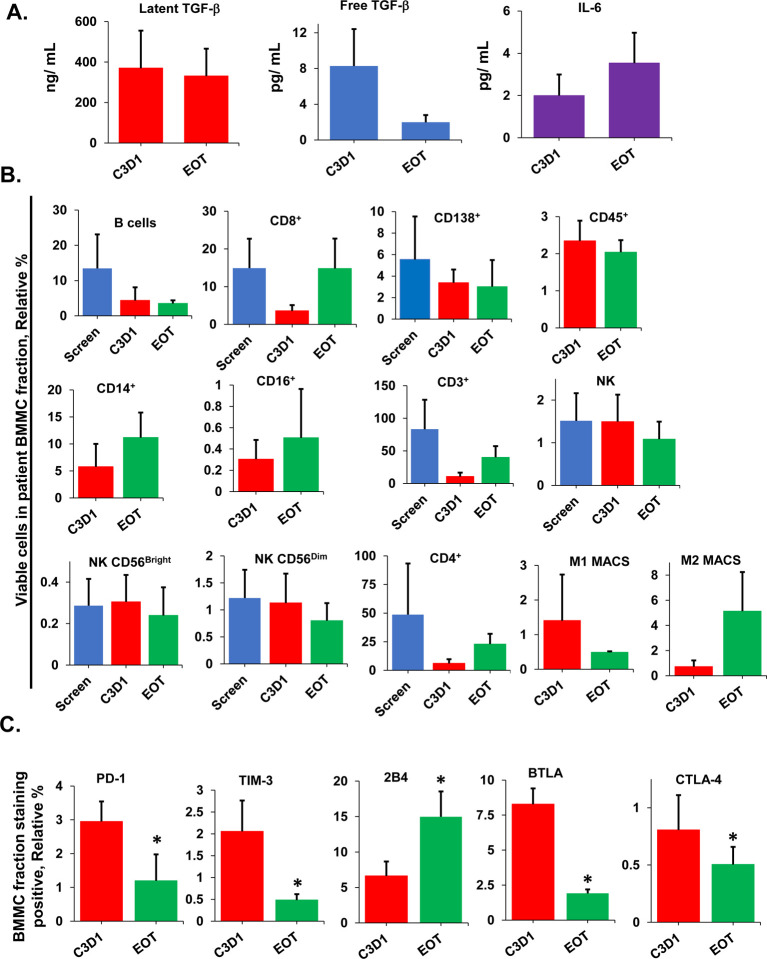
Effect of vactosertib and pomalidomide therapy on MM patient BM cytokines. **A.** Effect of vactosertib and pomalidomide on cytokines. At indicated times during treatment, latent TGF-β1, free (active) TGF-β1 and IL-6 levels were measured in BM samples from N ≥ 3 patients. Error bars represent the SD of the mean. **B.** Effect on vactosertib and pomalidomide on the relative percentage of tumor, immune and non-immune cell types. The mononuclear cell fraction was isolated from patient BM samples and stained using antibodies specific to B cells, CD4^+^ and CD8^+^ T cells, NK cells, leukocytes, monocytes, and macrophages in patient BM samples. Cell types were quantitated by immunostaining followed by flow cytometry. Cell types were quantitated from N ≥3 MM patients. Error bars represent the SD of the mean. **C.** Effect on vactosertib and pomalidomide on the relative surface expression of immunosuppressive markers on patient CD8^+^ T-cells. Relative levels of PD-1, TIM-3, 2B4, BTLA, and CTLA4 were measured at indicated times. Asterisks indicate statistical significance (*p* ≤ 0.05).

We next probed the systemic, tumor extrinsic effects of vactosertib by analyzing cytokines immune and tumor cells present within patient BM samples. Latent TGF-β1 levels were unchanged in patient BM during treatment and at the end of treatment (EOT), while IL6 levels were significantly higher in the same samples at the EOT (Fig. 4A). However, free (active) TGF-β1 levels were significantly reduced in patient BM samples at the EOT. Immunostaining followed by flow cytometry then indicated that B, CD38^+^/CD138^+^, CD3^+^, and M1 MACS cell numbers were decreased in patient BM samples following treatment, while CD14^+^, CD16^+^, M2 MACS and CD8^+^ cell numbers were increased (Fig. 4B). TGF-β1 has previously been shown to support T-cell development, homeostasis, tolerance, and differentiation leading to a functionally diverse T-cell pool (30–32). Therefore, we explored the immunophenotype of CD8^+^ T-cells isolated from patient BM prior to and at the EOT. The relative percentage of T-cells that expressed the immunosuppressive markers programmed cell death-1 (PD-1), T-cell immunoglobulin and mucin domain-containing protein 3 (TIM3), B and T lymphocyte attenuator (BTLA), and cytotoxic T-lymphocyte associated protein 4 (CTLA-4) was reduced following treatment relative to their expression prior to treatment (Fig. 4C). PD-1 is a member of the CD28 superfamily that delivers negative signals upon interaction with its ligands PD-L1 and PD-L2. PD-1 and its ligands are broadly expressed and exert immunoregulatory roles in T-cell activation (33). TIM3 is expressed on IFN-γ-producing CD4^+^ and CD8^+^ T-cells (34). BTLA is a CD28/B7 family member, that shares structural and functional similarity with CTLA-4 and PD-1 (35–38). Results indicated that vactosertib and pomalidomide treatment *in vivo* reduced PD-1, TIM3, BTLA and CTLA-4 expression on patient CD8^+^ T-cells.

**Figure 5.**
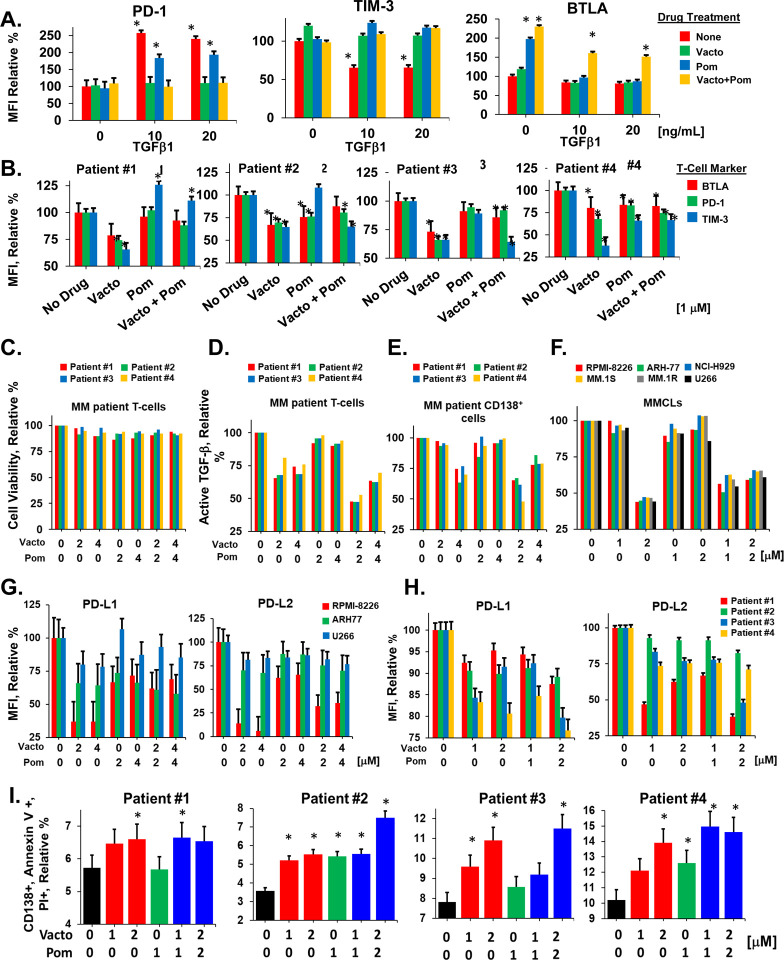
Effect of vactosertib and pomalidomide on CD8^+^ T-cell immunophenotype and functional activity. **A.** Shown is the relative MFI value for PD-1, TIM-3, BTLA, and LAG3 expression on CD8^+^ T-cells isolated from peripheral blood of healthy donors. T-cells were cultured in the presence of TGF-β1 at the indicated concentrations. Error bars represent the standard error of the mean. CTLA-4 was not detectable by immunostaining on healthy T-cells. Error bars represent the SD of the mean. **B.** Shown is relative MFI values after staining for PD-1, TIM-3, BTLA, and LAG3 on MM patient CD8^+^ T-cells. Error bars represent the SD of the mean. **C.** Effect of vactosertib on MM patient CD8^+^ T-cell viability. MM patient CD8^+^ T-cells were treated vactosertib and/or pomalidomide for 48 h. XTT assay solution was added to the plate and incubated in dark for 3 h. The relative cell viability was quantified by measuring the absorbance at 450_nm_ on SpectraMax i3x multi-mode microplate reader. **D.** Effect of vactosertib on TGFβ levels in patient CD8^+^ T-cell cultures. CD8^+^ T-cells were cultured with vactosertib, pomalidomide and combinations for 48 h. Cells were then centrifuged for 5 min at 3,000 × rpm. Supernatants were removed and TGF-β1 levels were determined using the human TGF-β1 duoSet ELISA kit. Error bars represent the SE of the mean. **E.** Effect of vactosertib on TGF-β1 levels in MMCL supernatants. Cells were cultured with vactosertib, pomalidomide and combinations as shown for 48 h. Cells were centrifuged for 5 min (3,000 × rpm), supernatants removed and TGF-β1 levels determined using the human TGF-β1 duoSet ELISA kit. **F.** Effect of vactosertib on TGF-β1 levels in patient CD138^+^ supernatants. Cells were cultured with vactosertib, pomalidomide and combinations for 48 h as shown, centrifuged for 5 min at 3,000 × rpm and supernatants removed. TGF-β1 levels were determined using the human TGF-β1 duoSet ELISA kit. **G.** Effect of vactosertib on PD-L1 and PD-L2 expression on MMCLs. MMCLs were treated with vactosertib and/or pomalidomide at indicated concentrations for 72 h. Cells were then stained with PD-L1 and PD-L2-specific antibodies simultaneously for 20 min and analyzed by flow cytometry. **H**. Effect of vactosertib on PD-L1 and PD-L2 expression on MM patient CD138^+^ cells. Patient CD138^+^ cells were treated with vactosertib and/or pomalidomide at various concentrations for 48 h. Cells were then stained with PD-L1 and PD-L2 specific antibodies simultaneously for 20 min and analyzed by flow cytometry. **I.** Effect of vactosertib on autologous T-cell cytotoxic activity. Shown is the cytotoxic effect of autologous CD8^+^ T-cells from patient peripheral blood on patient CD138^+^ cells following 24 h co-culture. CD138^+^ cells (20,000/well) were treated with drugs as indicated for 8 h and then co-cultured CD8^+^ T-cells (50,000/well) for 18 h. Cells were then stained with a CD138^+^-specific antibody, followed by propidium iodide and annexin-V for 15 min in the dark. Apoptosis was quantified in cells positively gated for CD138^+^, annexin-V and propidium iodide by flow cytometry using FlowJo_10.8.1 software. Black bars represent the effect of T-cells alone. Asterisks indicate statistical significance (*p* ≤ 0.05). Error bars represent the SD of the mean.

We next investigated the effect of vactosertib alone and with pomalidomide on CD8^+^ T-cells isolated from healthy donors and MM patients. The addition of physiologic doses of TGF-β1 to healthy CD8^+^ T-cells in culture increased PD-1 expression by 2.5-fold as indicated by immunostaining and flow cytometry (Fig. 5A). Treatment of healthy T-cells with TGF-β1 alone did not increase the expression of TIM3, BTLA or LAG-3 and CTLA-4 was not detectable on healthy T-cells. Healthy T-cells were then treated with vactosertib which suppressed the effect of TGF-β1 on PD-1 expression, when added alone or in combination with pomalidomide. The addition of pomalidomide alone slightly reduced PD-1 expression when added in the presence of TGF-β1. Pomalidomide alone or in combination with vactosertib, slightly increased BTLA and LAG-3 expression (Fig. 5B). Next, CD8^+^ T-cells were isolated from the peripheral blood of MM patients and cultured in the presence of TGF-β1, vactosertib, pomalidomide, or vactosertib plus pomalidomide (Fig. 5B). TGF-β1 did not increase the expression of PD-1 on patient CD8^+^ T-cells, most likely because these cells were isolated from the TGF-β1-rich TME of MM patient BM. Treatment of patient CD8^+^ T-cells with vactosertib decreased PD-1 expression up to 40% and decreased TIM3 expression nearly 65%. Pomalidomide alone did not significantly reduce PD-1, TIM3, BTLA, or LAG-3 on patient CD8^+^ T-cells. Vactosertib and pomalidomide also reduced TIM3 levels on CD8^+^ T-cells from most patients. Vactosertib, with or without pomalidomide, slightly decreased the viability of patient CD8^+^ T-cells but significantly reduced the amount of TGF-β1 produced in culture supernatants by ~50% (Fig. 5C, D). Vactosertib treatment *in vitro* also reduced the amount of TGF-β1 detected in culture supernatants from MMCLs and patient CD138^+^ cells (Fig. 5 E, F). Importantly, vactosertib treatment of MMCLs and patient CD138^+^ cells significantly reduced the surface expression of PD-L1 and PD-L2 (Fig. 5 G, H). Vactosertib reduced PD-L1 expression on MMCLs by 25–70% and PD-L2 expression by 20–80%. Similarly, vactosertib treatment reduced the level of PD-L1 detected on patient CD138^+^ cells up to 25% and the level of PD-L2 detected by nearly 60%. Patient CD138^+^ cells were then treated with vactosertib and pomalidomide and co-cultured with autologous CD8^+^ T-cells. Following co-culture, the percentage of annexin-positive CD138^+^ cells was quantitated by flow cytometry. Co-culture of patient CD138^+^ cells with autologous CD8^+^ T-cells generated a slight increase in percentage of annexin^+^ cells. Pre-treatment of patient CD138^+^ cells with vactosertib or vactosertib combined with pomalidomide followed by co-culture with CD8^+^ T-cells significantly increased the percentage of annexin^+^ cells (Fig. 5I).

## DISCUSSION

We report the first prospective analysis to demonstrate the safety, tolerability and efficacy of vactosertib and pomalidomide to treat RRMM, a population with an important unmet medical need. The patients included in our study are representative of a patient cohort with limited remaining treatment options. The proportion of patients that underwent previous ASCT (N=14, 67%) and the older age of patients included in the present study closely reflects the population of patients with RR disease. However, our results suggest that treatment with oral vactosertib in combination with pomalidomide was well-tolerated, even in transplant-ineligible and elderly patients. The overall response rate (ORR) of 32% (partial + minimal response) in efficacy-evaluable patients treated with vactosertib and pomalidomide exceeded ORRs reported for other agents evaluated to treat RRMM (39–41). For example, the first-in-class peptide-drug conjugate melphalan flufenamide (melflufen) targets aminopeptidases to rapidly and selectively release alkylating agents into tumor cells. Melphalan flufenamide plus dexamethasone demonstrated an ORR of 31% in the phase II HORIZON trial in RRMM patients (39). Similarly, in the phase 3 MM-003 study, the ORR after a median follow-up of 10·0 months was documented in 31% of patients treated with pomalidomide with low-dose dexamethasone (LoDEX) in RRMM patients (40). Lastly, selinexor plus dexamethasone resulted in objective treatment responses in patients with myeloma refractory to currently available therapies and a PR or better was observed in 26% of patients (41). Our results suggest that vactosertib leads to clinical improvement with potential long-term benefit for patients in whom other available therapies have not been effective. All patients included in the trial were resistant to proteasome inhibitors, and as our results demonstrate, combination of vactosertib with proteasome inhibitors overcame drug resistance. Vactosertib combined with bortezomib may represent a promising approach for transplant-ineligible and frail patients that are not eligible for current or emerging therapies.

The frequency and severity of adverse events were equal to or less than those observed with other regimens for RRMM. The most common toxicities and adverse events, e.g., fatigue, cough, pain, were infrequent, reversible and clinically manageable with dose reduction, dose delay and supportive care. Clinically significant bleeding, respiratory, cardiac, and infectious events were uncommon and non-hematological toxicities were infrequent. Permanent treatment discontinuation was very low (N=1) due adverse event. The proportion of secondary malignancies reported was zero, despite the inclusion of a significant number of patients on the study with a longstanding history of MM and had received an IMiD and alkylator exposure. Second primary malignancy in prior first-line, phase 3 studies has ranged from 2.5 to 10.7%, due to underlying disease and (potentially) treatment-related effects (42).

While corticosteroids have historically been the backbone of most myeloma-targeting therapies, their efficacy must be balanced with a considerable side-effect profile of acute and chronic toxicities (43–47). The presence of concomitant disease that requires treatment with high-dose corticosteroids represents a common exclusion criteria for immunotherapy trials (48). Dexamethasone, a key agent in myeloma induction regimens, is associated with side effects that disproportionately affect older patients. Newer agents with significant anti-myeloma activity now permit the development of steroid-free regimens. Despite the absence of steroids, the time to response did not appear significantly delayed with vactosertib since the median time to a first response was 1–2 months and median time for best response was 2 months. This compares favorably with previously reported steroid-containing regimens, e.g., bortezomib and thalidomide with or without dexamethasone, with a median time to response of ~1·4–2 months, (48, 49). Steroids impair T lymphocyte activation, reduce helper T-cell expansion, block regulatory T-cell recruitment and promote M2 macrophage polarization. The efficacy and favorable safety profile of vactosertib administered in steroid-sparing regimens may improve the treatment of elderly patients at greater risk of treatment toxicity and poor survival due to their age and comorbidities.

Cancers are characterized by an immunosuppressive TME that suppresses T-cell-mediated anti-tumor activity (50). PD-1 expression on T-cells serves as an immune checkpoint to attenuate downstream T-cell receptor (TCR) signaling and limit T-cell activation, while TGF-β1 directly inhibits effector T-cells (51). Therapeutic antibodies that target the PD-1/PD-L1 axis induce potent and durable anti-tumor responses in multiple cancer types but only in limited patient populations (52). As a negative regulator of anti-tumor immunity, TGF-β1 impairs the efficacy of anti-PD-1/PD-L1 directed therapy and promotes drug resistance. Our studies to characterize molecular mechanisms that govern T-cell-mediated treatment responses demonstrate that TGF-β1 promotes PD-1 expression on T-cells and that vactosertib suppresses the TGF-β1-driven upregulation of PD-1. Vactosertib also reduced PD-L1/PD-L2 on MM cells and enhanced T-cell activity. Our analysis is consistent with prior studies showing that exhausted T-cells are enriched within the TME but possess immunophenotypic and functional plasticity (53). Neoadjuvant immunotherapy to target immunosuppression offers advantages over upfront surgery and adjuvant therapy, including the potential to improve clinical outcomes and understand molecular mechanisms of treatment response and resistance (54). Vactosertib may serve a neoadjuvant role to block TGF-β1 signaling and revert the exhausted T-cell phenotype that precludes responses in RRMM and solid tumors.

Three drugs are now FDA-approved to target PD-1 (pembrolizumab, nivolumab and cemiplimab), three for PD-L1 (atezolizumab, avelumab and durvalumab). KEYNOTE-185 was a randomized, open-label, phase 3 trial for transplantation-ineligible NDMM patients. Treatment naive patients received either pembrolizumab plus lenalidomide and dexamethasone or lenalidomide and dexamethasone alone. However, an FDA-requested interim analysis showed that the benefit–risk profile of pembrolizumab with lenalidomide and dexamethasone was unfavorable for NDMM patients. Similarly, trials in MM patients with atezolizumab, pembrolizumab, nivolumab, durvalumab and avelumab were halted (55). Patients treated with these monoclonal antibodies receive continuous steroid therapy, either as part of treatment or to reduce immune-related adverse events that leads to immunosuppression and increased risk of infection. Systemic steroid use prior to starting immune checkpoint inhibitors also has a negative impact on survival.

Recent studies have harnessed the power of T-cells engineered to express chimeric antigen receptors (CARs) to treat MM and other hematologic malignancies (56–59). Barriers to effective CAR-T cell therapy include severe life-threatening toxicities, modest anti-tumor activity, antigen escape, restricted trafficking, and limited tumor infiltration. Furthermore, host and TME interactions critically alter CAR T-cell function, while a complex workforce is required to develop, expand and implement these treatments. Strategies to block TGF-β1 signaling and improve the next-generation of armored CAR T-cells, T-cell receptors (TCRs) and bispecific T cell engagers (BiTEs) remain in development. Vactosertib represents a safe, feasible approach to reduce immunosuppression, revert T-cell exhaustion and reinvigorate anti-myeloma immunity.

## Methods and Materials

### Cell lines.

Authenticated MMCLs RPMI8226, ARH77, U266, MM1.S, MM1.R, NCI-H929 were obtained from ATCC (Manassas, VA) and cultured under standard conditions in complete RPMI media that contained pen/strep and glutamine. Cell lines were tested for mycoplasma using the Mycostrip detection kit and then biobanked for future use. Cultures were replaced regularly from frozen stock. Saturated cultures were split 1:2 every 3 days.

### Chemicals.

Vactosertib, pomalidomide, lenalidomide, bortezomib, carfilzomib, and ixazomib, were from Selleck Chemicals (Houston, TX). DMSO, XTT tetrazolium salt (sodium 3′-[1-[(phenylamino)-carbony]-3,4-tetrazolium]-bis(4-methoxy-6-nitro)benzene-sulfonic acid hydrate), PMS (N-methyl dibenzopyrazine methyl sulfate) and general chemicals were from Sigma Chemical Co. (St. Louis, MO).

### Patient samples.

Patient samples were obtained and de-identified following IRB approval and informed consent. Peripheral blood samples were barcoded and stored at −80°C. BM samples were processed to isolate the mononuclear cell fraction by red blood cell lysis following by gradient centrifugation. CD138^+^ cells were isolated by positive selection using the EasySep^™^ human CD138 positive selection kit II (StemCell Technology, Cambridge, MA). CD8^+^ T-cells were isolated using the EasySep direct human CD8^+^ T-cell isolation kit (StemCell Technology).

### Antibodies.

To quantitate the relative level of individual cell types, patient BM-derived mononuclear cell fractions were stained with the following antibodies: FITC-conjugated anti-human CD19 antibody (Biolegend: 302205) to detect B cells, PE/Cyanine7-conjugated anti-human CD8 antibody (Biolegend: 344711) to detect CD8^+^ cells, FITC-conjugated anti-human CD38 antibody (Biolegend: 303504) to detect CD38^+^ cells, PerCP/Cyanine5.5-conjugated anti-human CD138 (Syndecan-1) antibody (Biolegend: 356509) to detect CD138^+^ cells, Pacific Blue^™^-conjugated anti-human CD45 antibody (Biolegend: 368540) to detect CD45^+^ cells, APC-conjugated anti-human CD14 antibody (Biolegend: 367118) to detect CD14^+^ cells, APC-conjugated anti-human CD16 antibody (Biolegend: 302012) to detect CD16^+^ cells, APC-conjugated anti-human CD3 antibody (Biolegend: 317318) to detect CD3^+^ cells, FITC-conjugated anti-human CD56 (NCAM) antibody (Biolegend: 318303) to detect NK cells, PE-conjugated anti-human CD80 antibody (Biolegend: 305207) to detect M1 MACS and FITC-conjugated anti-human CD163 antibody to detect M2 MACS. To quantitate the relative expression of immunosuppressive surface markers on CD8^+^ T-cells, cells were stained with antibodies specific to PD-1 (Biolegend: 329952), TIM-3 (Biolegend: 364806), LAG3 (Biolegend: 369343), BTLA (Biolegend: 344524) and CTLA-4 (Biolegend: 369606) according to manufacturer’s guidelines. PE-Cyanine7 conjugated anti-human CD138 (Syndecan-1) antibody (Invitrogen: 25–1389-41) was used to stain patient myeloma cells. Human normal peripheral blood CD8^+^ Cytotoxic T cells (HumanCells Biosciences, Milpitas, CA: PBCD-C10M) were treated with recombinant human TGFβ1 (HEK293-derived, Peprotech: 100–21) at 0, 5, 10, 20 and 30 ng/mL for 48 h and probed with antibodies specific to CTLA-4, PD-1, BTLA, TIM-3 and LAG-3. To quantitate relative PD-L1 and PD-L2 expression, MM patient cells and MMCLs were stained with FITC-conjugated anti-human CD274 (B7-H1, PD-L1) (Biolegend: 393606) and APC-conjugated anti-human CD273 (B7-DC, PD-L2) (Biolegend: 345508) according to manufacturer’s guidelines.

### Cytokine assays.

Cytokines were measured in freshly isolated patient BM samples using the human latency associated peptide (LAP) TGFβ1 Quantikine ELISA kit (catalog #: DLAP00), the human TGF-β1 duoSet ELISA kit (catalog #: DY240) and the human IL-6 Quantikine QuicKit ELISA (QK#206) from R&D systems (Minneapolis, MN).

### Flow cytometry.

Cells (10^6^/100uL) were stained with antibodies to markers in FACS staining buffer (1x PBS, 1% BSA, 0.1 % sodium azide) for 30 min at room temperature in the dark after which they were then washed thrice before analysis using a Life Technologies Attune NxT Flow Cytometer. Unstained cells and isotype-specific controls were included with each assay.

### Annexin/ propidium iodide staining.

MM cells were incubated as indicated treatments or co-cultured with CD8^+^ cytotoxic T-cells; where the treatment mediated, or T-cell mediated cell death was evaluated by flow cytometry for the detection of annexin-V and PI positive staining cells using the FITC Annexin V Apoptosis Detection Kit I (BD Biosciences, 556547).

### Assay method for measuring plasma concentration of vactosertib.

The plasma concentrations of vactosertib were quantified by liquid-chromatography multiple reaction monitoring (LC-MRM). Stock solutions of vactosertib (MedChemExpress, Monmouth Junction, NJ) and internal standard (IS, valsartan, MedChemExpress) were prepared at a concentration of 10 μg/mL in 0.1% formic acid. Serial dilution of vactosertib stock solution with 0.1% formic acid gave the corresponding working vactosertib solutions at concentrations of 390.625, 781.25, 1562.5, 3125, 6250, 12500, 25000, 50000, and 100000 ng/mL for the preparation of the calibration standards. Those solutions were further diluted 10-times with human plasma (Innovative Research, Novi, MI) and used as calibration standard samples. The plasma samples were thawed, and 10 μL of the IS stock solution was added to 10 μL of each sample. After a 10 s vortex, 80 μL of acetonitrile was added to each sample to precipitate proteins. The solution was then centrifuged at 14,000 g for 10 min. The supernatant was then transferred into a polypropylene insert (Agilent Technologies, Santa Clara, CA) placed in a HPLC autosampler vial for LC-MRM analysis as described below. LC-MRM analysis was carried out using an Agilent 1290 Infinity HPLC system (Agilent, Santa Clara, CA) coupled with Agilent 6460 Triple Quadrupole mass spectrometer equipped with an electrospray Jet Stream ion source. Following the sample preparation described above, 1 μL of sample was injected onto a Kinetex 2.6 μm Polar C18 100 Å (100 × 2.1 mm) column (Phenomenex, Torrance, CA) and chromatographed using a linear gradient of acetonitrile from 20% to 80% over 8 min in aqueous 0.1% formic acid at a flow rate of 200 μL/min. Mass spectrometric analysis employed electrospray ionization in the positive ion mode with MRM at the transitions of m/z 400.1 → 289.1 for vactosertib and 436.1 → 291.1 for IS. The optimized fragmentor voltage and collision energy for vactosertib were 140 and 13 V, respectively, and 84 and 17 V for IS. The optimized ion source parameters were as follows: capillary voltage (2000 V), Nozzle voltage (2000 V), nebulizer gas pressure (45 psi), sheath gas flow (11 L/min), and sheath gas temperature (300 °C). Nitrogen was used as the source and collision gas. Vactosertib and IS were eluted at 3.6 and 6.4 min, respectively. The developed LC-MRM method showed an excellent linearity between 39 – 5000 ng/mL with r2 > 0.99. The acquired LC-MRM data were analyzed by Agilent MassHunter Quantitative Analysis software to determine the plasma concentrations of vactosertib. Pharmacokinetic parameters were obtained from the plasma concentration data using Ubiquity pharmacokinetic modeling tool (60).

## Supplementary Material

1 **Supplementary Figure 1.** Shown is the disease level for each individual patient in trial determined at the beginning of each treatment cycle.
**Supplementary Figure 2.** Shown is the disease level for each individual patient in trial determined at the beginning of each treatment cycle.
**Supplementary Figure 3.** Shown is the scheme to generate RPMI8226 cells resistant to each FDA-approved proteasome inhibitor (bortezomib, carfilzomib and ixazomib).
**Supplementary Figure 4.** Shown is the relative effect of each PI on PI-sensitive and PI-resistant RPMI8226 cells. PI-resistant cells were generated as above and incubated in the presence of each PI at the indicated concentration for 72 h. Cell viability was determined using the XTT assay.

## Figures and Tables

**Table 1.** **Baseline characteristics of patients**.

**Sex**	Female	8 (38%)
	Male	13 (62%)
**Race**	White	13 (62%)
	Black/AA	7 (33%)
	Asian	1 (5%)
	Other	0 (0%)
**Age, years**	Median	68 (range 55–77)
**ECOG Score**		
	0	6 (29%)
	1	15 (71%)
**Disease type**	IgG	15 (71%)
	IgA	5 (24%)
	IgM	0 (0%)
	LC	1 (5%)
**ISS Score at diagnosis**		
	1	3 (14%)
	2	8 (38%)
	3	7 (33%)
	Unknown	3 (14%)
**Cytogenetic risk at initial diagnosis**		
	High	5 (24%)
	Standard	16 (76%)
**Prior systemic lines of therapy**		
	1	3 (14%)
	2	12 (57%)
	3	4 (19%)
	4	2 (10%)
**Prior drug theraies**		
	Proteasome Inhibitor	21 (100%)
	IMiD	21 (100%)
	Melphalan	14 (67%)
**Previous ASCT**		14 (67%)
**Median prior lines of therapy**		2
**Best response to previous treatment**		
Stable disease		9 (36%)
Minimal/Marginal response		2 (11%)
Partial response		4 (21%)
Progressive disease		4 (21%)
Not evaluable		2 (10%)

Summary of baseline characteristics by demographics, prior treatment regimen and prior response to therapy. Cytogenetic risk was considered high based upon the t(4;14), t(14;16) and t(14;20) translocations that have been associated with poor prognosis, and their presence identifies high-risk (HR) disease. On the other hand, patients with t(11;14), t(6;14) and/or trisomies are considered to have standard-risk (SR) disease. Data are presented as n (%).

**Table 2.** Summary of adverse events.

	Grade 1–2	Grade 3	Grade 4	Grade 5
**Blood and lymphatic disorders**				
ALT increased	3 (15%)	2 (10%)	0 (0%)	0 (0%)
AST	2 (10%)	2 (10%)	0 (0%)	0 (0%)
Alkaline phosphatase increased	0 (0%)	1 (5%)	0 (0%)	0 (0%)
Anemia	16 (79%)	2 (10%)	0 (0%)	0 (0%)
Blood bilirubin increased	2 (10%)	2 (10%)	1 (5%)	0 (0%)
Lymphopenia	15 (72%)	6 (29%)	0 (0%)	0 (0%)
Neutrophil count decreased	2 (57%)	11 (52%)	0 (0%)	0 (0%)
WBC decreased	16 (76%)	2 (10%)	0 (0%)	0 (0%)
Febrile neutropenia	0 (0%)	1 (5%)	0 (0%)	0 (0%)
**Coagulation**				
Epistaxis	2 (10%)	0 (0%)	0 (0%)	0 (0%)
INR increased	1 (5%)	0 (0%)	0 (0%)	0 (0%)
**Dermatologic**				
Bullous dermatitis	1 (5%)	0 (0%)	0 (0%)	0 (0%)
Maculopapular rash	8 (39%)	2 (10%)	0 (0%)	0 (0%)
Pruritus	13 (62%)	0 (0%)	0 (0%)	0 (0%)
Skin and cutaneous tissue disorders	2 (10%)	0 (0%)	0 (0%)	0 (0%)
Skin hyperpigmentation	1 (5%)	0 (0%)	0 (0%)	0 (0%)
**Gastrointestinal disorders**				
Abdominal pain	3 (15%)	0 (0%)	0 (0%)	0 (0%)
Diarrhea	1 (5%)	0 (0%)	0 (0%)	0 (0%)
Constipation	5 (24%)	0 (0%)	0 (0%)	0 (0%)
Gastroesophageal reflux	1 (5%)	0 (0%)	0 (0%)	0 (0%)
Gastrointestinal disorders	0 (0%)	0 (0%)	0 (0%)	0 (0%)
Lipase increase	0 (0%)	0 (0%)	0 (0%)	0 (0%)
Nausea	4 (19%)	0 (0%)	0 (0%)	0 (0%)
Pancreatitis	0 (0%)	1 (5%)	0 (0%)	0 (0%)
Serum amylase increased	2 (10%)	1 (5%)	0 (0%)	0 (0%)
Vomiting (0%)	4 (19%)	0 (0%)	0 (0%)	0
**General disorders and administration site condition**				
Anorexia	2 (10%)	0 (0%)	0 (0%)	0 (0%)
Chills	1 (5%)	0 (0%)	0 (0%)	0 (0%)
Cough	9 (43%)	0 (0%)	0 (0%)	0 (0%)
Dry mouth	1 (5%)	0 (0%)	0 (0%)	0 (0%)
Dysgeusia	3 (15%)	0 (0%)	0 (0%)	0 (0%)
Fall	3 (15%)	0 (0%)	0 (0%)	0 (0%)
Fatigue	12 (57%)	0 (0%)	0 (0%)	0 (0%)
Flu-like symptoms	1 (5%)	1 (5%)	0 (0%)	0 (0%)
Gait disturbance	1 (5%)	0 (0%)	0 (0%)	0 (0%)
Generalized muscle weakness	3 (14%)	2 (10%)	0 (0%)	0 (0%)
Headache	4 (19%)	0 (0%)	0 (0%)	0 (0%)
Hyperhidrosis	1 (5%)	0 (0%)	0 (0%)	0 (0%)
Malaise	1 (5%)	0 (0%)	0 (0%)	0 (0%)
Pain	8 (39%)	1 (5%)	0 (0%)	0 (0%)
Pain in extremity	10 (47%)	1 (5%)	0 (0%)	0 (0%)
Palpitations	2 (10%)	0 (0%)	0 (0%)	0 (0%)
Post-nasal drip	1 (5%)	0 (0%)	0 (0%)	0 (0%)
Productive cough	3 (14%)	0 (0%)	0 (0%)	0 (0%)
Pyrexia	2 (10%)	0 (0%)	0 (0%)	0 (0%)
Sneezing	1 (5%)	0 (0%)	0 (0%)	0 (0%)
Sore throat	1 (5%)	0 (0%)	0 (0%)	0 (0%)
Tinnitus	2 (10%)	0 (0%)	0 (0%)	0 (0%)
Productive cough	3 (14%)	0 (0%)	0 (0%)	0 (0%)
Pyrexia	2 (10%)	0 (0%)	0 (0%)	0 (0%)
Sneezing	1 (5%)	0 (0%)	0 (0%)	0 (0%)
Sore throat	1 (5%)	0 (0%)	0 (0%)	0 (0%)
Tinnitus	2 (10%)	0 (0%)	0 (0%)	0 (0%)
**Infections and infestations**				
Lung infection	1 (5%)	0 (0%)	0 (0%)	0 (0%)
Salivary gland infection	0 (0%)	1 (5%)	0 (0%)	0 (0%)
Upper respiratory tract infection	3 (15%)	0 (0%)	0 (0%)	0 (0%)
Other	1 (5%)	0 (0%)	0 (0%)	0 (0%)
**Metabolism and nutritional disorders**				
Hypercalcemia	2 (10%)	0 (0%)	0 (0%)	0 (0%)
Hypercalcemia	2 (10%)	0 (0%)	0 (0%)	0 (0%)
Hypoalbuminemia	2 (10%)	0 (0%)	0 (0%)	0 (0%)
Hypocalcemia	7 (33%)	0 (0%)	0 (0%)	0 (0%)
Hypokalemia	4 (20%)	0 (0%)	0 (0%)	0 (0%)
Hypomagnesemia	1 (5%)	0 (0%)	0 (0%)	0 (0%)
Hyponatremia	6 (29%)	0 (0%)	0 (0%)	0 (0%)
Hypophosphatemia	3 (15%)	0 (0%)	0 (0%)	0 (0%)
**Musculoskeletal and connective tissue disorders**				
Back pain	7 (34%)	0 (0%)	0 (0%)	0 (0%)
Chest wall pain	1 (5%)	0 (0%)	0 (0%)	0 (0%)
Musculoskeletal and				
Connective tissue disorder-other	2 (10%)	0 (0%)	0 (0%)	0 (0%)
Musculoskeletal deformity	1 (5%)	0 (0%)	0 (0%)	0 (0%)
Neck pain	1 (5%)	0 (0%)	0 (0%)	0 (0%)
Flank pain	2 (10%)	0 (0%)	0 (0%)	0 (0%)
**Cardiac disorders**				
Atrial fibrillation	2 (10%)	0 (0%)	0 (0%)	0 (0%)
AV block- first degree	1 (5%)	0 (0%)	0 (0%)	0 (0%)
Cardiac troponin I increased	1 (5%)	0 (0%)	0 (0%)	0 (0%)
Sinus bradycardia	5 (24%)	0 (0%)	0 (0%)	0 (0%)
**Vascular**				
Edema (face)	1 (5%)	0 (0%)	0 (0%)	0 (0%)
Edema (limbs)	2 (10%)	0 (0%)	0 (0%)	0 (0%)
Edema (localized)	2 (10%)	0 (0%)	0 (0%)	0 (0%)
Hypotension	1 (5%)	0 (0%)	0 (0%)	0 (0%)
Hypertension	4 (19%)	2 (10%)	0 (0%)	0 (0%)
Syncope	0 (0%)	1 (5%)	0 (0%)	0 (0%)
**Nervous system disorders**				
Blurred vision	2 (10%)	0 (0%)	0 (0%)	0 (0%)
Concentration impairment	1 (5%)	0 (0%)	0 (0%)	0 (0%)
Confusion	1 (5%)	0 (0%)	0 (0%)	0 (0%)
Dizziness	8 (38%)	0 (0%)	0 (0%)	0 (0%)
Extraocular paresis	1 (5%)	0 (0%)	0 (0%)	0 (0%)
Eye disorders, other	1 (5%)	0 (0%)	0 (0%)	0 (0%)
Peripheral sensory neuropathy	1 (5%)	0 (0%)	0 (0%)	0 (0%)
**Pulmonary**				
Dyspnea	7 (33%)	0 (0%)	0 (0%)	0 (0%)
Nasal congestion	3 (15%)	0 (0%)	0 (0%)	0 (0%)
Respiratory, thoracic and Mediastinal disorders	1 (5%)	0 (0%)	0 (0%)	0 (0%)
Sleep apnea	1 (5%)	0 (0%)	0 (0%)	0 (0%)
Wheezing	1 (5%)	0 (0%)	0 (0%)	0 (0%)
**Hepatic**				
Cholecystitis	1 (5%)	0 (0%)	0 (0%)	0 (0%)
**Orthopedic**				
Fracture	1 (5%)	0 (0%)	0 (0%)	0 (0%)
**Renal**				
Creatinine increase	5 (24%)	0 (0%)	0 (0%)	0 (0%)
Renal calculi	1 (5%)	0 (0%)	0 (0%)	0 (0%)

Treatment-emergent adverse events of any grade, which were reported in at least 5% of patients in the treatment group are shown. Data are presented as n (%).

**Table 3.** Best overall response to therapy.

	Vactosertib and Pomalidomide
**Response**	**N (%)**
Partial response	4 (21%)
Minimal/Marginal response	2 (11%)
Stable disease	9 (47%)
Progressive disease	4 (21%)
Not evaluable	2 (11%)
**Overall Response Rate** (Partial + Minimal)	6 (32%)
**Clinical Benefit Ratio (CBR)** (Stable disease + Partial + Minimal)	15 (69%)

Data are n (%), unless stated otherwise. Overall response to treatment (secondary endpoint) was derived according to IMWG criteria.
