# Supplementary material for: Vactosertib, a novel TGF-β1 type I receptor kinase inhibitor, improves T-cell fitness: a single-arm, phase 1b trial in relapsed/refractory multiple myeloma
Source: Res Sq. 2023 Jul 17:rs.3.rs-3112163. Preprint. [Version 1] doi: 10.21203/rs.3.rs-3112163/v1 (PMC10371138; doi:10.21203/rs.3.rs-3112163/v1)
Supplement: 1 — Supplementary Figure 1. Shown is the disease level for each individual patient in trial determined at the beginning of each treatment cycle. Supplementary Figure 2. Shown is the disease level for each individual patient in trial determined at the beginning of each treatment cycle. Supplementary Figure 3. Shown is the scheme to generate RPMI8226 cells resistant to each FDA-approved proteasome inhibitor (bortezomib, carfilzomib and ixazomib). Supplementary Figure 4. Shown is the relative effect of each PI on PI-sensitive and PI-resistant RPMI8226 cells. PI-resistant cells were generated as above and incubated in the presence of each PI at the indicated concentration for 72 h. Cell viability was determined using the XTT assay. [file NIHPPRS3112163V1-supplement-1.pdf]

Supplementary Table 1. Pharmacokinetic parameters of vactosertib in RRMM patients.

	Mean ± SD			
**Dose**	C_{max} **(ng/mL)**	T_{max} **(h)**	*t* **½ (h)**	AUC_{obs} **(ng/mL·h)**
100 mg (n = 2)	1785.7 (± 559.8)	1.3 (±0.5)	3.5 (±0.4)	6175.5 (±1349.7)
200 mg (n =4)	996.7 (± 503.7)	1.5 (±1.0)	3.3 (±1.3)	4251.8 (±1307.6)

Supp. Fig. 1

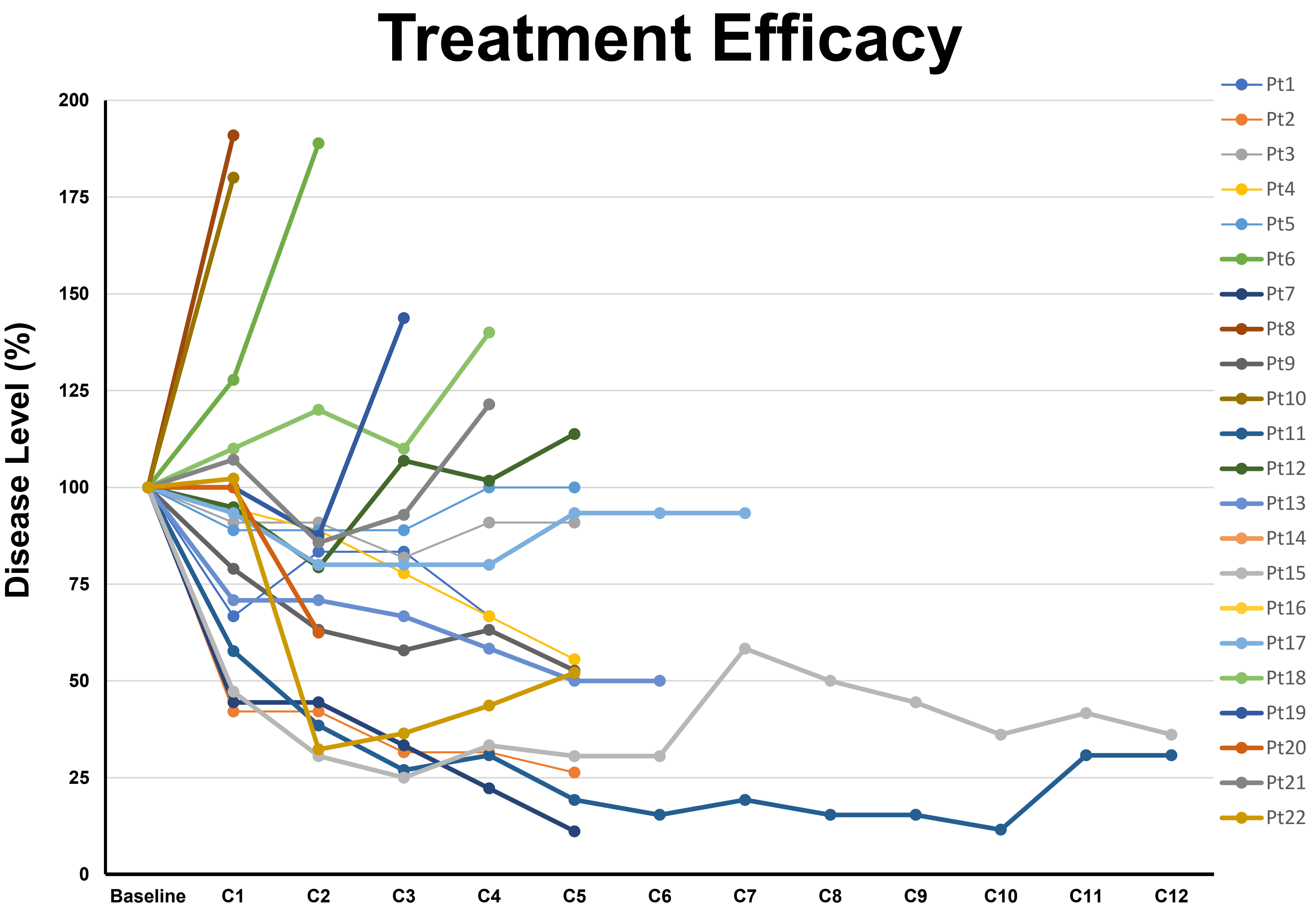
Treatment Efficacy
Disease Level (%)
200
175
150
125
100
75
50
25
0
Baseline
C1
C2
C3
C4
C5
C6
C7
C8
C9
C10
C11
C12
Pt1
Pt2
Pt3
Pt4
Pt5
Pt6
Pt7
Pt8
Pt9
Pt10
Pt11
Pt12
Pt13
Pt14
Pt15
Pt16
Pt17
Pt18
Pt19
Pt20
Pt21
Pt22

Supp. Fig. 2

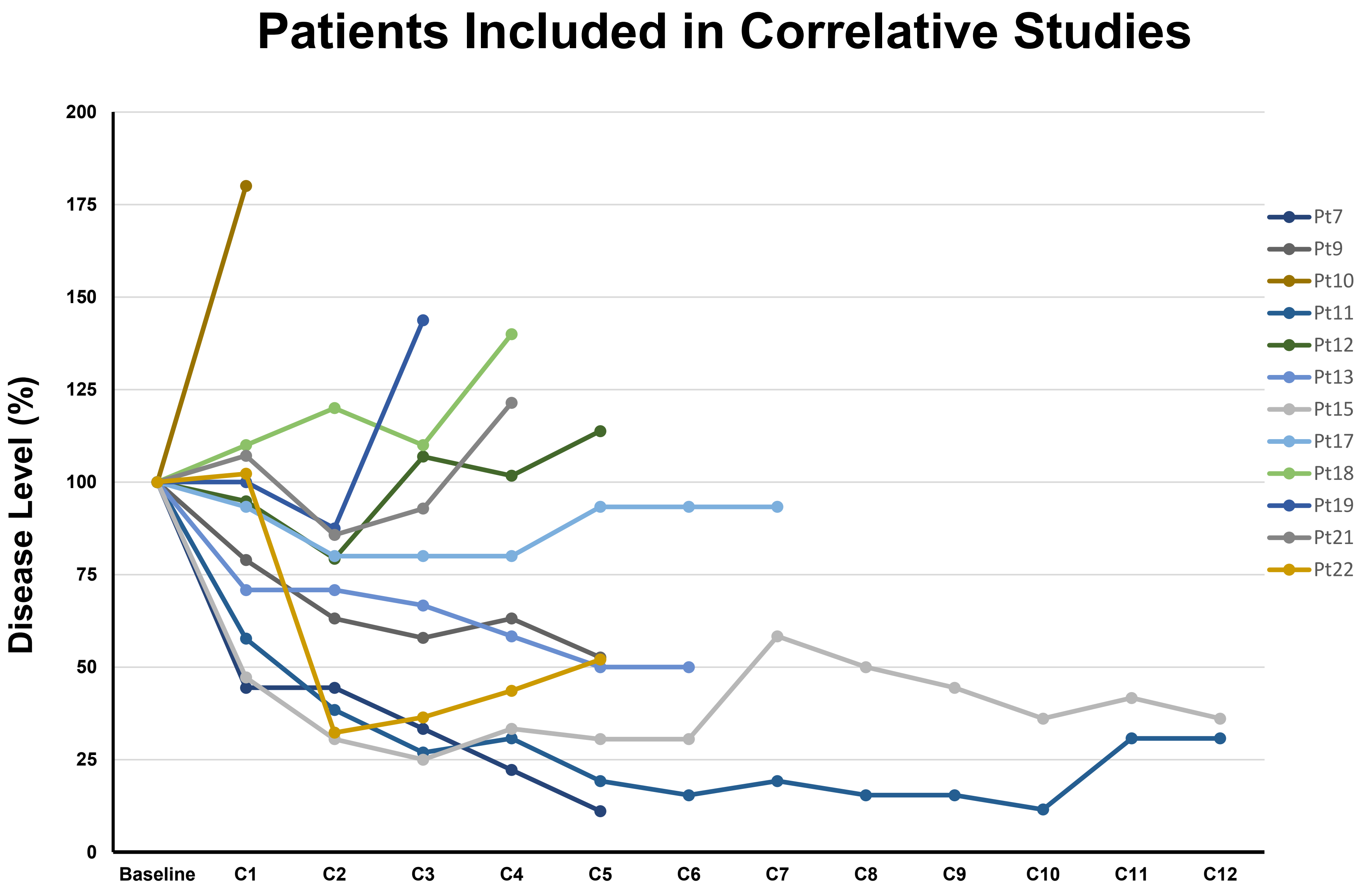
Patients Included in Correlative Studies
Disease Level (%)
0
25
50
75
100
125
150
175
200
Baseline
C1
C2
C3
C4
C5
C6
C7
C8
C9
C10
C11
C12
Pt7
Pt9
Pt10
Pt11
Pt12
Pt13
Pt15
Pt17
Pt18
Pt19
Pt21
Pt22

Supp. Fig. 3

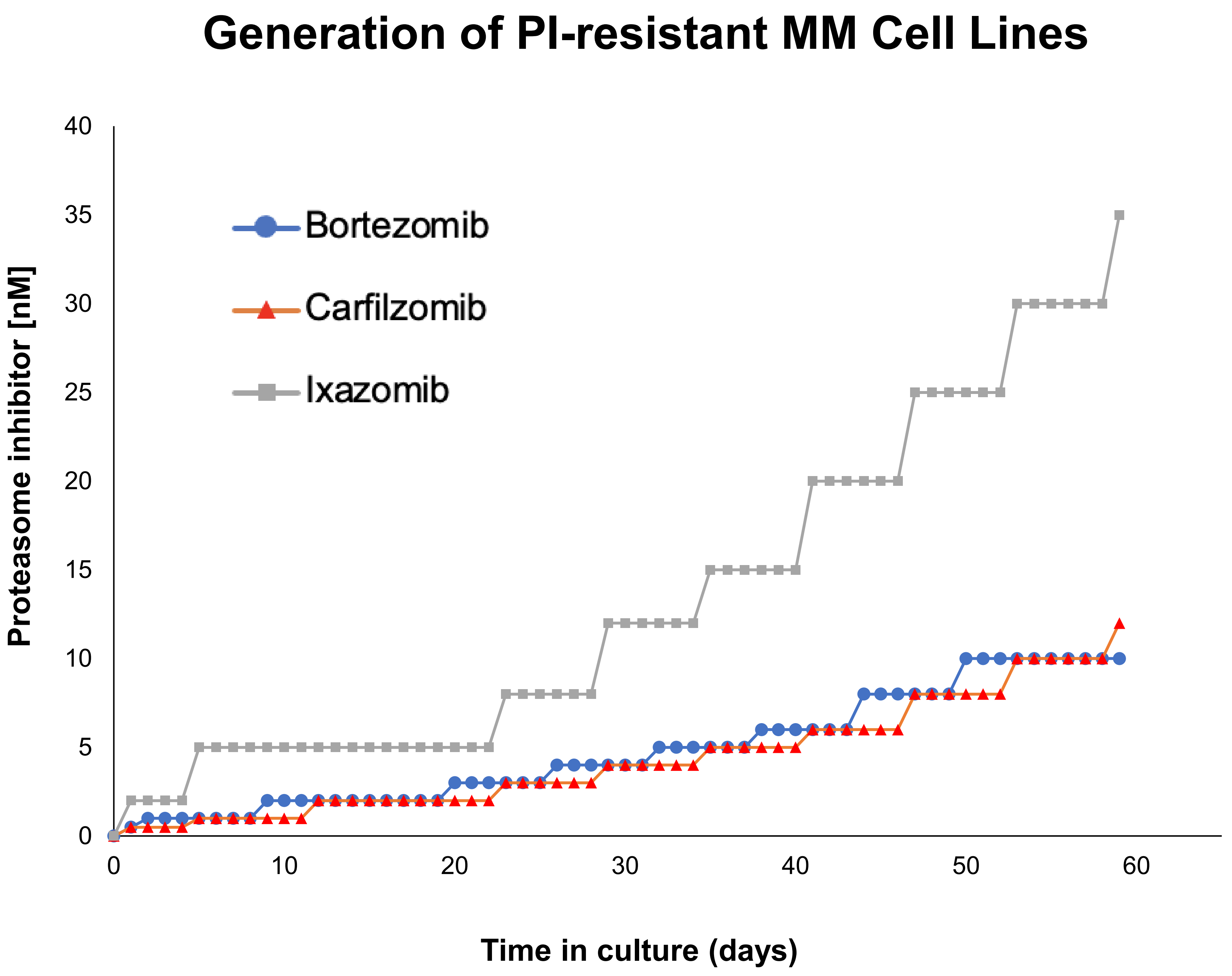
Generation of PI-resistant MM Cell Lines
Bortezomib
Carfilzomib
Ixazomib
Proteasome inhibitor [nM]
Time in culture (days)
0
5
10
15
20
25
30
35
40
0
10
20
30
40
50
60

Supp. Fig. 4

Cross-Resistance of MM Cell Lines to Proteasome Inhibitors

Cell Viability, Relative %

Cell Line	PI Tested	0	1	3	5	10	25	50	75	100	200
Parental	Bortezomib	100.00	100.00	78.27	29.79	0.00	0.00	0.00	0.00	0.00	0.00
BTZ-R	Bortezomib	100.00	98.28	100.00	100.06	99.60	60.29	28.17	10.99	15.29	16.26
CFZ-R	Bortezomib	100.00	88.11	91.46	78.88	92.12	69.26	24.51	6.64	2.75	0.00
IXZ-R	Bortezomib	100.00	100.00	96.58	99.71	94.93	100.00	100.00	4.47	0.00	0.00
Parental	Carfilzomib	100.00	91.58	70.75	32.66	0.00	0.00	0.00	0.00	0.00	0.00
BTZ-R	Carfilzomib	100.00	86.94	82.53	71.94	71.53	75.13	5.40	8.01	18.09	10.39
CFZ-R	Carfilzomib	100.00	75.95	76.02	92.64	80.75	98.45	20.04	6.23	1.31	0.00
IXZ-R	Carfilzomib	100.00	100.00	89.76	100.00	93.51	99.56	77.52	0.00	7.84	0.00
Parental	Ixazomib	100.00	100.00	100.00	100.00	100.00	32.72	0.00	0.00	0.00	0.00
BTZ-R	Ixazomib	100.00	80.17	71.19	87.10	87.02	98.25	83.81	46.55	1.47	2.68
CFZ-R	Ixazomib	100.00	99.77	100.00	100.00	92.86	93.03	50.76	39.13	11.46	0.00
IXZ-R	Ixazomib	100.00	100.00	98.43	79.37	72.02	38.07	38.76	5.68	19.53	16.36

PI Concentration, nM

APPENDIX

CLINICAL TRIAL AGREEMENT for INVESTIGATOR-INITIATED STUDY

THIS AGREEMENT is by and between **Medpacto**, a corporation organized under the laws of the Republic of Korea, whose offices are located at 145 Gwanggyo-ro, AICT Building C, 5th Floor, Room 506, Suwon, 16229, Republic of Korea (hereinafter referred to as "Company"); and **University Hospitals Cleveland Medical Center**, a non-profit academic medical center, whose address is 11100 Euclid Avenue, Cleveland, Ohio 44106 (hereinafter referred to as "Institution").

Scope of Work. Institution will use reasonable efforts to perform the experiments and studies described in the investigator-initiated protocol entitled, **"A Phase I Trial of TEW-7197 in Combination with Pomalidomide (POM) in Relapsed or Relapsed and Refractory Multiple Myeloma (RRMM)"** incorporated herein by reference (hereinafter referred to as the "Study"). Company acknowledges that the primary mission of Institution is health care, education, and the advancement of knowledge, and consequently, all services provided by Institution under this Agreement will be performed in a manner best suited to carry out that mission. Institution does not guarantee specific results of the Study.

1. **Principal Investigator.** The Study will be performed under the direction of Ehsan Malek, MD (hereinafter referred to as "Principal Investigator"). Principal Investigator will conduct and supervise the Study in accordance with the aforementioned protocol, this Agreement, the investigational plan, and any and all applicable laws, regulations (including FDA regulations), and conditions of approval imposed by the IRB or the FDA, and will comply with all requirements regarding the obligations of clinical investigators and all pertinent requirements thereunder, including 21CFR312. Principal Investigator will also ensure that all associates, colleagues, and employees of Institution assisting in the conduct of the Study are informed about their obligations to Company per this Agreement. In the event the Principal Investigator is unable or unwilling to continue with the Study, the party first learning of this inability or unwillingness will notify the other party in writing and the parties will attempt to find a mutually acceptable substitute. In the event a mutually acceptable substitute is not found, the Agreement may be terminated in accordance with Paragraph 10.

2. **Company Obligations.**

    2.1 In performing its obligations under this Agreement, each party will comply with, and will cause its affiliates, employees, and agents to comply with, the requirements of all applicable laws, rules, and regulations, including, without limitation, The Social Security Act, the Stark Law, the Anti-Kickback Statute, the False Claims Act, the Health Insurance Portability and Accountability Act (HIPAA), and all other applicable state and federal laws, rules and regulations, as each of the foregoing may be amended from time to time.

    2.2 Company warrants and represents that it is not excluded, nor otherwise been determined to be ineligible to participate in the Federal health care programs or in Federal procurement or nonprocurement programs (collectively, "Ineligible"). Sponsor agrees to notify Institution immediately, in the event Company becomes Ineligible. Upon receipt of such notification Institution shall have the right to terminate this Agreement immediately upon notice to Company.

2.3 Company agrees to promptly notify the Principal Investigator and the IRB both during the study and for two (2) years after the completion of the Study at all sites of any information, including but not limited to, (i) non-compliance with the Protocol or applicable laws (ii) serious adverse events, (iii) unanticipated problems, (iv) Study results or findings, in each case that could affect subjects' safety, subjects' willingness to continue to participate in the Study, or the IRB's continuing approval of the Study.

2.4 Company represents and warrants that the Study Drug is manufactured in accordance with good manufacturing procedures and will be labeled and shipped appropriately marked containers in accordance with 21 C.F.R. § 312.6.

3. **Term.** The term of this Agreement is from the effective date of May 2, 2017. The term of this Agreement may be extended by a written amendment signed by the authorized representative of both parties.

4. **Reports.** Upon completion of the research or termination of the Agreement, Institution will submit a written study report. This report will be due one-hundred twenty (120) days after termination of this Agreement.

5. **Free Product.** Company agrees to provide Institution Study Drug, **TEW-7197** in support for the Study.

6. **Equipment.** Title to any equipment purchased by Institution in the performance of the Study whether or not purchased with funds provided under this Agreement will remain with Institution and will be free of all claims, liens, or encumbrances of the Company.

7. **Publications.** Company acknowledges that the free dissemination of information is an important policy of Institution. Nothing herein shall prevent Institution or Principal Investigator from using such Study Data for ordinary, non-commercial research and educational purposes of a Institution, including its own publication, presentation, and instructional objectives, provided that the publication, presentation or use does not disclose Confidential Information furnished by Sponsor. Institution agrees that any proposed publication or presentation relating to the Study conducted under this Agreement will be submitted to Company for review at least thirty (30) days prior to submission for publication or presentation to remove Confidential Information. As such, the scope of Confidential Information in this publication context does not include the results arising out of the performance of this Agreement. In the event that the proposed publication or presentation contains patentable subject matter which needs protection, Institution will, upon written request from Company within the initial thirty (30) day review period, delay the publication or presentation for a maximum of an additional ninety (90) days to allow Company or Institution to file a patent application.

8. **Compensation:** Compensation for this Study shall be as outlined in the budget, attached hereto as .

8. **Intellectual Property.**

8.1. As this Agreement is for an investigator-initiated Study, Institution will promptly disclose to Company, in confidence, all creative ideas, developments and inventions, whether or not patentable, conceived or first reduced to practice as a result of the Project ("Inventions"). Institution hereby grants to Company the first option for a worldwide, exclusive, royalty-bearing license to make, have made, use, and sell with a right to sublicense, Inventions. The terms of such license will be reasonable in the circumstances and will be negotiated in good faith between the Company and Institution. The option to license any Invention will extend for a time period of six (6) months from the date of its original disclosure to Company.

8.2. Title to, and the right to determine the disposition of, any copyrights or copyrightable material, first produced or composed in the performance of this Study, shall remain with the Institution.

9. **Confidentiality and Record Retention.**

9.1. Institution acknowledges that Company may, prior to and during the term of this Agreement, provide Institution with scientific, technical, trade secret, business, or other information which is treated by Company as confidential or proprietary (hereinafter referred to as "Confidential Information"). In recognition that Institution is a non-commercial, academic institution, Company agrees to limit to the extent possible the delivery of confidential information to Institution. Both parties agree that in order to ensure that each party understands which information is deemed to be confidential, all Confidential Information will be in written form and clearly marked as "Confidential," and if the Confidential Information is initially disclosed in oral or some other non-written form, it will be confirmed in writing and clearly marked as "Confidential" within thirty (30) days of disclosure. Institution shall hold such Confidential Information in strict confidence and shall treat such information in the same manner as it treats its own confidential information. The Institution retains the right to refuse to accept any such information or data from Company which it does not consider to be essential to the completion of the Study or which it believes to be improperly designated. The Confidential Information provided to Institution by Company will remain the property of the Company, and will be disclosed only to those persons necessary for the performance of this Agreement. No indirect or consequential damages or damages based on loss of profits or market share are contemplated or recoverable for breach of confidentiality.

9.2. The obligation of Institution to maintain the Confidential Information under this Agreement will survive its expiration or termination and will endure for five (5) years from the date of disclosure.

9.3. The obligation of non-disclosure will not apply to any part of the Information that:

(a) is already known to Institution prior to the effective date, as evidenced by Institution's records;

(b) becomes publicly known without the wrongful act or breach of this Agreement by Institution;
(c) has been or is disclosed to Institution by a third party who was not, or is not, under any obligation of confidence or secrecy to Company at the time said third party discloses to Institution, or has the legal right to do so;
(d) is developed independently by employees of Institution who had no access to or knowledge of the Information, as evidenced by Institution's records;
(e) is approved for release by written authorization of the Company;
(f) is required to be disclosed by law or governmental regulation or to any governmental entity with jurisdiction, provided Institution promptly notifies Company, if reasonably practical or possible, in writing of such lawful disclosure.

9.4. If Company provides Institution with any proprietary study drugs and/or devices for use under the Study, such Company proprietary materials will be used solely for the Study and not for any other purposes. Institution and Principal Investigator shall be responsible for compliance with all laws and regulations applicable to any destruction or disposition of Company proprietary materials used under the Study. Institution and Principal Investigator will inform all potential Study participants that the proprietary study drugs and/or devices are being used for investigational purposes. Prior to using any proprietary study drugs and/or devices of Company, Principal Investigator shall read and understand all information in the investigator's brochure, including the potential risks and side effects of the drug. Upon completion or termination of this Study, Institution shall return, at Company's expense, or destroy any remaining Company proprietary materials at the direction and request of Company.

10. **Termination.**

10.1. Provisions of this Agreement which by their nature contemplate rights and obligations of the parties to be enjoyed or performed after the expiration or termination of this agreement for any reason will not relieve either party of its obligations under this Agreement previous to the effective date of such termination.

10.2. In the event that either party defaults or breaches any material provision of this Agreement, the other party may terminate this Agreement upon thirty (30) days written notice to the party in default or breach; provided, however, that if the party defaulting, breaching, or failing, within thirty (30) days of the receipt of such notice cures the said default, breach or failure; the Agreement will continue in force and effect.

10.3. This Agreement may be terminated by either party, upon immediate prior notice, if the authorization and approval to perform the Study in the United States is withdrawn by the FDA or, if the emergence of any adverse reaction or side effect with the drug administered or the device employed in the Study is of such magnitude or incidence in the opinion of either the Company or the Institution to support termination.

10.4. If either party should become insolvent or should make any assignment for the benefit of creditors, or should be adjudged bankrupt, or should file a petition in bankruptcy, or is named as debtor in an involuntary bankruptcy proceeding, or if a receiver or trustee of the property of either party is appointed, then this Agreement, at the option of the other party, will terminate, effective on the date notice of such termination is given.

10.5. Should Company terminate this Agreement, Company will reimburse Institution for all expenditures and non-cancelable commitments incurred prior to termination not to exceed the total amount of USD $446,582 of the Agreement.

11. **Assignment.** Neither party may assign this Agreement or any part of it without the written consent of the other party.

12. **Indemnification.**

12.1. The Company will defend, indemnify and hold harmless the Principal Investigator, the Institution, its affiliated hospitals and institutes, and their trustees, officers, employees, agents, and third parties acting on its/their behalf or with its/their authorization (hereafter collectively referred to as "Indemnitees") from any and all suits, actions, claims, demands, judgments, costs or liabilities, including attorneys fees and court costs at the trial and appellate levels, for any loss, damage, injury, or loss of life arising from the manufacture of any study drugs or devices provided by Company to Institution for use in the Study, or arising from products and tangible items developed or made as a result of information or materials received from the Institution, provided that (i) Institution promptly notifies Company in writing after Institution receives notice of any claim, (ii) Company is given the opportunity, at its option, to participate and associate with Institution in the control, defense and trial of any claim and related settlement negotiations.

13. **Publicity.**

The parties agree that neither party will use the names or trademarks of the other party, nor any adaptation thereof in any advertising, promotional or sales activities without prior written consent obtained from the other party.

Company acknowledges that the names and affiliations of Company and the general purposes and budget of the Study are to be made public by Institution to satisfy its reporting obligations or as required by law or regulation.

14. **Compliance with HIPAA and Use of Study Data.**

Institution agrees to comply with all applicable state and federal laws and regulations, including the Health Insurance Portability and Accountability Act of 1996, as codified at 42 U.S.C. § 1320d ("HIPAA") and any current and future regulations promulgated

thereunder. Both parties agree that the use of data generated under this Study shall be governed by the terms and conditions of the Informed Consent and HIPAA authorization forms, which have been or will be approved by Institution's IRB. Terms and conditions of this Agreement shall not supersede or modify the use of data terms and conditions listed in the Informed Consent and HIPAA authorization forms. Principal Investigator will ensure that the requirements relating to and obtaining Informed Consent and IRB review and approval are met.

15. Disputes.

Both parties shall work together in good faith in attempt to resolve any dispute arising under this Agreement. Any dispute or proceeding under this Agreement shall be subject to the jurisdiction and venue of the courts of the State of Ohio, United States of America or the United States Federal courts having jurisdiction in Ohio, and both parties hereby consent to the personal jurisdiction and venue of these courts.

16. Independent Contractor.

Nothing contained herein will be construed as establishing an employer-employee, joint venture, or principal-agent relationship between the parties. In addition, neither party will have the right to incur any debt or expense for the account of the other party except as may expressly be agreed upon by separate written agreement.

17. Miscellaneous.

The headings in this Agreement are intended solely for convenience or reference and will be given no effect in the construction or interpretation of this Agreement.

This Agreement, including attached appendices, supersedes all prior oral and written proposals and communications, if any, and sets forth the entire Agreement of the parties with respect to the subject matter hereof and may not be altered or amended except in writing, signed by an authorized representative of each party hereto. The terms in this Agreement take precedence over the protocol.

The construction and enforcement of this Agreement will be governed by the laws of the State of Ohio, United States of America, without regard to principles of choice of law. The parties acknowledge that this contract is entered into and will be performed in Ohio.

No waiver of any default, condition, provision or breach of this Agreement will be deemed to imply or constitute a waiver of any other like default, condition, provision or breach of this Agreement.

If any paragraph, term, condition or provision of this Agreement will be found, by a court of competent jurisdiction, to be invalid or unenforceable, or if any paragraph, term, condition or provision is found to violate or contravene the laws of the State of Ohio, then the paragraph, term condition or provision so found will be deemed severed from

this Agreement, but all other paragraphs, terms, conditions and provisions will remain in full force and effect.

18. **Notices.** Notices to be provided between the parties shall be provided to the following individuals for each party:

COMPANY:

MedPacto, Inc.
145 Gwanggyo-ro
AICT Building C, 5th Floor, Room 506
Suwon, 16229
Republic of Korea
Attn: Administration Director

INSTITUTION:

University Hospitals Cleveland Medical Center
11100 Euclid Avenue
Cleveland, OH 44106
Attn: UH Clinical Research Center

With a copy to:

University Hospitals Cleveland Medical Center
3605 Warrensville Center Road
Shaker Heights, OH 44122
Attn: Chief Legal Officer

<Signatures on the following page>

IN WITNESS WHEREOF, the parties hereto have caused this Agreement signed by their respective officers duly authorized as the date and year written.

**University Hospitals Cleveland Medical Center**	**MedPacto, Inc.**
Signed: ____________ Date: 05-09-17	Signed: ____________ Date: 5/11/2017
Name: Daniel I. Simon, MD	Name: Seong Jin Kim
Title: President, UHCMC	Title: CEO

University Hospitals APPROVED AS TO FORM
Abigail M. Blanks
UH Law Department
2017.05.02
13:55:43 -04'00'

READ AND ACKNOWLEDGED:

PRINCIPAL INVESTIGATOR

Signed: ____________ Date: 5/9/2017

Name: Ehsan Malek, MD

Exhibit A

University Hospitals Cleveland Medical Center
PI: Dr. Ehsan Malek
CASE 1A17
Date: 4/25/2017

Invoiceable Costs	2017 Rate
UH SCC Continuing Review, Amendment, or Study Closure Application Preparation and Submission (No Consent Change)	$1,000
UH SCC Continuing Review, or Amendment Application Preparation and Submission (With Consent Change)	$1,200
IRB Fee - Continuing Review, Amendment, or Study Closure	$1,000
IRB Fee - Continuing Review or Amendment with Consent Chnages	$1,200
Re-Consenting Fee (per patient)	$200
Screen Failure	see budget
Pharmacy Annual Renewal	$1,000
Pharmacy Close Out & Drug Disposal Fee	$500
Administrative Maintenance Annual Fee	$500
Record Retention Annual Fee	$500
Each SAE Report, UH Subjects	$250
Outside IND Safety Letters (each)	$30
Clinical Trial Agreement Revisions	$750
Study Closeout Administrative Fee	$500

Startup Fees	2017 Rate
Admin Startup Fee	$7,000
PRMC Startup Fee	$500
SCC IRB Prep & Submission - initial review	$2,000
IRB Initial Review	$2,000
Compilion eReg Fee	$2,000
TRC Startup Fee	$500
Investigational Pharmacy Startup Fee	$2,000
CCRT Startup	$1,000
**Total Other Costs**	**$17,000**

CASE1A17 Protocol Version Date: 4-21-2017			Cycle 1					Cycle 2				Cycle 3				Cycle 4				Cycle 5				Cycle 6				
Cycles: 28/21 Days Max Cycles:6	CPT Code	Screen	D1	D5	D8	D15	D22	D1	D8	D15	D22	D1	D8	D15	D22	D1	D8	D15	D22	D1	D8	D15	D22	D1	D8	D15	D22	End of Tx
Informed Consent	N/A	X																										
Background Information	N/A	X																										
Inclusion/exclusion Criteria	N/A	X																										
Bone Marrow Examination (archived sample)	38221	X																										
Vital Signs	N/A	X	X		X	X	X	X				X				X				X				X				X
Medical History	N/A	X	X	X	X	X	X	X				X				X				X				X				X
Physical Examination	99214	X	X	X	X	X	X	X				X				X				X				X				X
Blood Draw	36415	X	X		X	X	X	X	X	X	X	X	X	X	X	X	X	X	X	X	X	X	X	X	X	X	X	X
CBC w/Diff	85025	X	X		X	X	X	X	X	X	X	X	X	X	X	X	X	X	X	X	X	X	X	X	X	X	X	X
Comprehensive Panel	80053	X	X		X	X	X	X	X	X	X	X	X	X	X	X	X	X	X	X	X	X	X	X	X	X	X	X
Magnesium	83735	X	X		X	X	X	X	X	X	X	X	X	X	X	X	X	X	X	X	X	X	X	X	X	X	X	X
PT/INR	85610	X																										
APTT	85730	X																										
TSH, T3 Free, T4 Free	84443, 84481, 84439	X														X												
Pregnancy Test: Serum	84702	X						X				X				X				X				X				
Cardiac Enzymes (Creatinine Kinase & Troponin)	82550, 84484	X	X		X	X	X	X	X	X	X	X	X	X	X	X	X	X	X	X	X	X	X	X	X	X	X	
Quantitative Serum Immunoglobulin Levels (Local Lab)	82784x3, 82785, 82784	X						X				X				X				X				X				
Serum protein electrophoresis (SPEP) and 24-hour Urine electrophoresis (UPEP) (Local Lab)	84165,86334 and 84166,86335	X						X				X				X				X				X				
Serum free light-Chain (FLC) assay (Local Lab)	83883x2	X						X				X				X				X				X				
ECG	93005/93010	X	X	X	X	X	X	X		X		X		X		X		X		X		X		X		X		
ECHO	93308	X						X								X								X				X
Skeletal Survey	77075	X																										X
Compliance Review/Drug Calendar	N/A	X						X				X				X				X				X				X
IDS: TEW-7197 (PO)	IDS		X	X	X	X	X	X	X	X	X	X	X	X	X	X	X	X	X	X	X	X	X	X	X	X	X	
Pomalidomide (PO)			X	X	X	X		X	X	X		X	X	X		X	X	X		X	X	X		X	X	X		
Total Per Visit Per Patient		$2,410	$1,996	$582	$1,021	$1,021	$1,021	$1,680	$383	$634	$383	$1,076	$383	$634	$383	$1,680	$383	$634	$383	$1,076	$383	$634	$383	$1,680	$383	$634	$383	$1,321

Per Patient Totals	
Total Cost Per Patient	$23,560
**Patient Related Totals**	
Total Number of Patients	18
Total Cost All Patients	$424,082

Fixed Costs		
Startups Total	$	17,000.00
Minimum Annual Invoiceable Items	$	3,000.00
Closeouts Total	$	2,500.00
TOTAL FIXED COSTS	$	22,500.00
Total Study Costs		$446,582

CLINCIAL TRIAL PAYMENT INFORMATION

University Hospitals Cleveland Medical Center

1. CLINICAL TRIAL INFORMATION

**Site Name:**	University Hospital Cleveland Medical Center
**Principal Investigator:**	Ehsan Malek, MD
**Study No.:**	CASE 1A17
**Protocol Title:**	A Phase I Trial of TEW-7197 in Combination with Pomalidomide (POM) in Relapsed or Relapsed and Refractory Multiple Myeloma (RRMM)
**IND #**	134775
**Number of Patients to be Enrolled:**	18

2. PAYMENT INFORAMTION

A. All of the study related and site costs are included in Exhibit A in US dollars.

B. The Per Patient Visit Cost includes non-standard of care study-related costs for each patient as required in the Protocol, including procedure costs, site personnel fees, administrative fees during performance of the study, lab draws and overhead. The Per Patient Visit Costs section of the budget represents procedures required to be performed on every patient.

C. Screen Failures for randomized patients who do not complete the study, the Site will be paid according to the per visit schedule noted in Exhibit A for those completed Screening visit documented by electronic data capture or other approved data input.

D. Site Invoiceable Costs will be paid upon 45 days of receipt of an invoice with documentation

E. Startup Costs ($17,000) Payment will be made upon agreement execution.

F. Payments will be made in quarterly installments based on the invoiced number of completed visits per randomized patient. Patient visit data is obtained in-house according to information provided by the Electronic Data Capture System (EDC). The sites will monitor the study data and monitoring visit outcomes will be made available to MedPacto Inc. for review.

G. All payments notifications should be emailed to UHCRCGA&RFSTeamsms@UHhospitals.org

3. INVOICE SUBMISSON

A. Please direct all invoices to the Sponsor Payment Coordinator assigned to this study. All invoices will be emailed to: Insun Baek

MedPacto, Inc.
145 Gwanggyo-Ro,
AICT Building C, Room 506
Suwon, 16229, Republic of Korea

Telephone: +82-70-8610-2727
Fax: +82-31-888-9949
Email: insun.baek@medpacto.com

B. All invoices will include: Site Name, PI Name and Study Number
C. All invoices will be paid upon 45 days of receipt of an invoice.

4. PAYMENT INFORMATION

A. All payments will be in USD Currency
B. The Site's Tax Identification Number is 34-1567805
C. All payments notifications should be emailed to UHCRCGA&RFSTeamsms@UHhospitals.org
D. Each ACH Payment will be made payable to University Hospitals Cleveland Medical Center will reference the Protocol study number CASE1A17 and submitted to

ACH PAYMENT

Bank: JPMorgan Chase

Routing: 044000037

Account: 209622228

5. CONTACTS

University Hospitals Cleveland Medical Center
11100 Euclid Avenue
Cleveland, OH 44106
Attn: UH Clinical Research Center

Technical Matters:

Dr. Ehsan Malek, Principal Investigator
Ehsan.Malek@uhhospitals.org

Administrative Matters:

Laura Baker, Budget Analyst II
Laura.Baker@uhhospitals.org

MedPacto Inc
Room 506, 5F, Building C
Advanced Institutes of Convergence Technology
145 Gwanggyo-ro
Suwon, 16229, Republic of Korea

Agreement Matters:

Byungjoon Min, Administration Director
mbj@medpacto.com

Administrative Matters:

Insun Baek, Manager
insun.baek@medpacto.com
